# Neandertal versus Modern Human Dietary Responses to Climatic Fluctuations

**DOI:** 10.1371/journal.pone.0153277

**Published:** 2016-04-27

**Authors:** Sireen El Zaatari, Frederick E. Grine, Peter S. Ungar, Jean-Jacques Hublin

**Affiliations:** 1 Paleoanthropology, Senckenberg Center for Human Evolution and Paleoenvironment, Eberhard Karls Universität Tübingen, Tübingen, Germany; 2 Department of Human Evolution, Max Planck Institute for Evolutionary Anthropology, Leipzig, Germany; 3 Department of Anthropology, Stony Brook University, Stony Brook, New York, United States of America; 4 Department of Anatomical Sciences, Stony Brook University, Stony Brook, New York, United States of America; 5 Department of Anthropology, University of Arkansas, Fayetteville, Arkansas, United States of America; Université de Poitiers, FRANCE

## Abstract

The Neandertal lineage developed successfully throughout western Eurasia and effectively survived the harsh and severely changing environments of the alternating glacial/interglacial cycles from the middle of the Pleistocene until Marine Isotope Stage 3. Yet, towards the end of this stage, at the time of deteriorating climatic conditions that eventually led to the Last Glacial Maximum, and soon after modern humans entered western Eurasia, the Neandertals disappeared. Western Eurasia was by then exclusively occupied by modern humans. We use occlusal molar microwear texture analysis to examine aspects of diet in western Eurasian Paleolithic hominins in relation to fluctuations in food supplies that resulted from the oscillating climatic conditions of the Pleistocene. There is demonstrable evidence for differences in behavior that distinguish Upper Paleolithic humans from members of the Neandertal lineage. Specifically, whereas the Neandertals altered their diets in response to changing paleoecological conditions, the diets of Upper Paleolithic humans seem to have been less affected by slight changes in vegetation/climatic conditions but were linked to changes in their technological complexes. The results of this study also indicate differences in resource exploitation strategies between these two hominin groups. We argue that these differences in subsistence strategies, if they had already been established at the time of the first contact between these two hominin taxa, may have given modern humans an advantage over the Neandertals, and may have contributed to the persistence of our species despite habitat-related changes in food availabilities associated with climate fluctuations.

## Introduction

Over the course of the last half million years, western Eurasian hominins lived during times of extreme climatic instability characterized by high amplitude fluctuations between cold glacial and warmer interglacial phases. These fluctuations constantly shaped and reshaped the landscape, greatly affecting both plant and animal communities [1]. Paleolithic hominins relied on these communities for subsistence, and therefore would have had to adapt to the frequent and sometimes dramatic, multisecular scale changes in dietary resources in order to survive. The occupation of Europe by members of the Neandertal lineage for hundreds of thousands of years amidst these continuously changing conditions suggests that they had effective subsistence strategies that allowed them to survive for such a lengthy period. Yet, it appears that during the severe millennial scale climatic fluctuations of Marine Isotope Stage (MIS) 3 [2], the survival strategies of the Neandertals failed, perhaps in part due to competition with modern humans who first entered Europe during this period [3]. Although a small amount of introgression of Neandertal DNA is documented in early modern Eurasians [4–6] and such an introgression is still detectable in extant non-African humans [7], there was undoubtedly a major population replacement in MIS 3. It seems counterintuitive that Neandertals, who had been living in Europe for such a long time and had managed to overcome earlier climatic cycles, disappeared, leaving the invading modern humans to flourish. Simply stated, Neandertals might be expected to have been better adapted than *Homo sapiens*—a species that evolved in Africa—to live in Europe during the fluctuations of MIS 3. But, the replacement of Neandertals by modern humans suggests that the latter may have had some advantages over the former.

Here, we present evidence for differences in dietary responses to climatic changes between Neandertals and modern humans in western Eurasia, which most likely gave the latter a survival advantage. This evidence takes the form of variation in dental microwear textures, and ergo diet, that accompanied fluctuations in ecological conditions. We employ a large sample of Paleolithic individuals (n = 52) that encompasses their geographical and temporal ranges (i.e., from 37 sites spread across western Eurasia that range in age from ca. 500 to 12 ka). We also present previously unpublished occlusal molar microwear data for 11 Middle Pleistocene specimens (S1 Table and S1 File) that date to between MIS 12–11 (e.g., Arago) and MIS 7 (e.g, Biache-Saint-Vaast). We here refer to this group as “early Neandertals” since Neandertal traits are expressed in all of the included individuals, albeit to varying degrees [8–10]. In addition, we summarize and reanalyze previously published data for later Neandertals [11–13] and for Upper Paleolithic modern humans [14] (S1 Table), and we offer the first comparison of the microwear textures among these three hominin groups.

Microwear texture analysis presents a proxy for the inference of dietary variations among Paleolithic hominin groups. The link between dental microwear textures and the mechanical properties of ingested items has been well-established through the examination of a variety of extant mammal species with well-documented diets [15–18]. Several surface texture attributes—complexity, anisotropy, texture fill volume, scale of maximum complexity, and heterogeneity—have proven useful for the overall characterization of diets. Complexity, measured as change in surface roughness with the scale of observation, has been considered a proxy for food hardness. Anisotropy, a measure of surface texture orientation, has been associated with toughness. Textural fill volume, a measure of size and depth of wear features, and scale of maximum complexity, the scale at which roughness increase tails off, have both been related to the sizes of abrasive particles. Finally, heterogeneity, or variability in complexity across the surface, is likely related to variation in food properties. These microwear texture attributes have been shown to discriminate samples by diet and to be especially valuable for detecting intra-species dietary differences, reflecting subtle differences in food preferences and dietary breadth [15, 18–20]. Since microwear signatures are dynamic with a high turnover rate capturing an individual’s diet a short time (i.e., weeks to months) prior to death, their analysis has the potential to give insights into the effects of environmental and technological changes on the diets of early and late Neandertals and Upper Paleolithic modern humans.

Such insights are crucial for documenting any differences in subsistence strategies between these hominin groups. Even though differences in subsistence strategies between Neandertals and modern humans have often been considered to have played a major role in their respective fates, the nature of such differences remains poorly understood. Middle Paleolithic faunal assemblages have led some researchers to argue that Neandertals focused on large ungulates whereas Upper Paleolithic humans broadened their dietary spectrum to include smaller mammals and aquatic resources [21–23]. However, various archaeological and geochemical analyses have shown that this view cannot be generalized to all Neandertal populations since at least some groups, especially those that lived in the warmer southern/Mediterranean regions, appear to have also exploited a diversity of terrestrial as well as possible marine resources [11, 24–29]. Stable isotope analyses have also contributed to the argument that modern humans had a broader dietary spectrum compared to Neandertals based on the former’s higher δ15N values, which have been linked to freshwater resource consumption [30–31]. Bocherens and colleagues [32], however, have recently cautioned that this observed enrichment could simply be due to a global enrichment in nitrogen-15 in terrestrial trophic webs between 31–35 ka cal BP rather than a dietary shift. However, the modern human remains from Ust’-Ishim (Siberia), which date to around 45 ka cal BP and thus predate this time period, already display high δ15N values [5]. As a whole, the picture of subsistence strategies of Neandertals and modern humans appears to be more complex than can be explained by a simple expansion of the dietary spectrum at the Middle to Upper Paleolithic transition. Since both of these hominin groups occupied vast geographic regions offering different food resources that also changed through time in response to climatic fluctuations, an understanding of intraspecific dietary variation in response to these fluctuations is necessary before comparing subsistence strategies among the Paleolithic hominin taxa. Equally important is the understanding of the possible role the Upper Paleolithic technological complexes played in helping modern human adapt to climatic instability. This study explores these aspects in a sample of Paleolithic individuals that encompasses the wide temporal and geographical ranges they occupied.

## Materials and Methods

Occlusal molar microwear texture data collected from a total of 52 pre-MIS 6 European hominins, MIS 6–3 Neandertals, and MIS 3–2 modern humans from 37 sites are included in this study (S1 Table). Microwear signatures for 11 pre-MIS 6 European individuals are analyzed here (see the Acknowledgements section for the repository information of the original specimens). Neandertal and modern human microwear texture data were obtained from published sources [11–14]. All necessary permits were obtained prior to the described study, which complied with all relevant regulations.

High-resolution dental casts for all specimens were prepared following established protocols [33]. After cleaning the teeth with cotton swabs soaked in distilled water—and in acetone and/or ethyl alcohol as needed—molds were made with President MicroSystemTM (Coltène-Whaledent) regular body impression material. Positive casts were then poured using Epo-Tek 301 epoxy resin and hardener (Epoxy Technology). Using a Sensofar Plμ Confocal Imaging Profiler (Solarius Development, Inc.), four adjoining scans, covering a total area of 276 x 204 μm of crushing/grinding “Phase II” facets of one molar per individual were taken at 100x magnification, with a lateral sampling interval of 0.18 μm and vertical resolution specification of <0.005 μm. The scans were analyzed with Toothfrax and SFrax software (Surfract) to generate the five variables mentioned above that describe different aspects of the surface textures: complexity (*Asfc*), anisotropy (*epLsar*), scale of maximum complexity (*Smc*), textural fill volume (*Tfv*), and heterogeneity (*HAsfc*). Detailed descriptions of these variables and their computations can be found in Scott et al. [34–35] and El Zaatari [15]. Values for *Asfc*, *epLsar*, *Smc*, and *Tfv* obtained from the four scans per specimen were used to calculate median values for each variable which were used to represent each specimen. The *HAsfc* value for each specimen was measured as the median absolute deviation of *Asfc* divided by the median of *Asfc* for the four scans representing each individual specimen without further splitting single scans into smaller sub-regions.

To assess the relationship between climatic change and microwear for the Paleolithic hominins each specimen included in this study was first assigned to one of three paleoecological categories—open, mixed, or wooded—based on all available paleoclimatic/paleoenvironmental data from the same sites and levels/layers that yielded the hominin individuals (see S1 File for details). In the context of Pleistocene western Eurasia, and as is evident from the paleoecological reconstructions summarized in S1 File, the open category represents the cold-steppe biome where open vegetation prevailed over the landscape and trees were relatively scarce, not exceeding 10% of the overall vegetation and/or where faunal records show a strong dominance of taxa that preferred cold, open habitats in the assemblage. The wooded habitats, on the other hand, correspond to woodlands where arboreal vegetation formed more than 40% and dominated over open vegetation and/or where woodland adapted animal taxa dominated the faunal assemblages. All specimens attributed to the wooded category in this study lived in forests that developed in warm climates and generally sustained deciduous as well as Mediterranean arboreal taxa (see S1 File). Finally, the mixed habitats represent intermediate environments that included significant amounts of both open and wooded vegetation elements, with percentages of trees ranging between 10% and 40%, and/or with faunal records showing a mix of taxa including species that preferred both open and wooded habitats. It should be acknowledged that the mixed category used in this study is relatively broadly defined and could potentially encompass a wide range of ecological zones where combinations of different open and wooded species would have covered the landscape. Mixed habitats in southern and Mediterranean Europe and the Levant would have been dominated by warm-loving Mediterranean taxa. But, with increasing latitude, the Mediterranean taxa would have gradually given way to deciduous and coniferous trees during temperate conditions as is evident from the pollen spectra of the various central European sites (e.g., Montmaurin, Saint-Césaire, La Chaise, Dolní Věstonice) with mixed vegetation (see S1 File). Unfortunately, the very small sample sizes that would result from splitting the mixed category into several sub-divisions dictated the use of a single broad mixed habitat category in this study although it is acknowledged that this might undermine the power of statistical tests in detecting significant differences between this group and those from wooded and especially open paleoecological categories.

It should also be acknowledged that the paleoecological reconstructions for the Upper Paleolithic deposits yielding the specimens included in this study show that the sampled specimens lived either in open or mixed habitats and that none are associated with wooded habitats (S1 Table). To our knowledge, there are no well-preserved molars from Upper Paleolithic adult individuals from wooded habitats. This is not surprising considering the generally cold conditions that prevailed in western Eurasia from the second half of MIS 3 to the end of MIS 2 and that maintained vegetation cover largely open in character. Indeed, even during the warmer interstadials of MIS 3, high densities of trees were recorded only in few places in Eurasia, namely around the Mediterranean [1]. Otherwise, open coniferous/deciduous woodland vegetation covered most of the southern European landscape, open coniferous woodlands covered parts of central Europe, whereas steppe vegetation prevailed further north [1]. Thus, for the Upper Paleolithic humans, assessments of links between diet and paleoecological conditions were restricted to those involving two habitat types, open and mixed. At the same time, however, these modern human individuals could be assigned to one of three technological complex categories (i.e., Aurignacian, Gravettian, Magdalenian) based on the available descriptions of their associated archaeological assemblages (S1 File).

After the assignment of individuals to the appropriate technological and/or paleoecological category, non-parametric Spearman’s rho and Kendall’s tau correlation coefficients were employed to test for correlations between diet and paleoecological conditions for each of the three groups of Paleolithic hominins (i.e., the early Neandertals, later Neandertals, and modern humans) and also between diet and technological developments through time for the Upper Paleolithic group. For the Neandertals, the microwear data for the five variables were compared against three paleoecological ranks (open = rank 1, mixed = rank 2, wooded = rank 3), whereas for the Upper Paleolithic humans these data were compared against two paleoecological ranks (open = rank 1, mixed = rank 2) and three technological complex ranks (Aurignacian = rank 1, Gravettian = rank 2, and Magdalenian = rank 3).

To assess differences in the five microwear variables among groups from different paleoecological settings within each of the Neandertal samples, a one-way multivariate analysis of variance (MANOVA) was conducted [36]. Yet, for the Upper Paleolithic humans, since two grouping factors—paleoecology and technology—were used, a two-way MANOVA was conducted to examine differences in microwear signatures among the different modern samples. This two-way MANOVA was run twice while replacing the missing paleoecological information for Labatut 1 with either of the two possible reconstructions: open or mixed habitats. It should be noted that it is highly unlikely that wooded conditions prevailed during the time of deposition of the layer containing this individual since this stratum is attributed to the Gravettian (Upper Perigordian) and since, as discussed above, vegetation in Europe appears to have remained relatively open during this time period of MIS 3. Thus, the results of the MANOVA model run with this third possibility are not reported here. However, it is still worth noting that such an assignment does not significantly alter the MANOVA results. Finally, to assess differences in diet, and thus patterns of resource and niche exploitation between Neandertals and earlier (i.e., Aurignacian and Gravettian) modern humans in Europe, a one-way MANOVA was used to compare the data for the five microwear variables between the earlier modern humans and their Neandertal counterparts from similar open and mixed environmental settings. For the purposes of this analysis, individuals attributed to both early and later Neandertals were grouped together and those associated with Aurignacian and Gravettian industries were grouped together to increase sample size. This resulted in four groups: Neandertals from either open or mixed categories, and earlier modern humans from either open or mixed categories.

All microwear data were rank transformed to mitigate violation of assumptions associated with parametric statistical tests before conducting the MANOVA analyses. The MANOVA analyses were followed by single classification ANOVA’s and two post-hoc tests, Tukey’s HSD (honestly significant difference) and Fisher’s LSD (least significant difference) tests to determine sources of significant differences when present [37–38]. These two post-hoc tests were used to balance Type I and II errors [37–38]. The null hypotheses for all these statistical tests state that the samples from different paleoecological categories and/or technological complexes have similar microwear textures.

## Results

Analyses of the data show that within the Neandertal lineage, dental microwear signatures are significantly associated with paleoecological conditions (Tables 1–3). Specifically, increase in tree cover is associated with an increase in microwear surface complexity (Tables 1 and 2). Early as well as later Neandertals from wooded habitats evince significantly higher complexity values than their counterparts from mixed and open habitats (Table 3) and later Neandertals from mixed habitats also possess marginally significantly higher complexity values than those from open habitats (Table 3). In addition, among late Neandertals, heterogeneity is also positively correlated with tree cover (Table 1).

**Table 1:** Correlation results for microwear variables and paleoecological and technological complexes ranks for the Paleolithic hominins.

		*Asfc*	*epLsar*	*Smc*	*Tfv*	*HAsfc*
Early Neandertals	Spearman’s rho	***0*.*810***	0.072	-0.114	0.111	0.530
open: n = 2, mixed: n = 2, wooded: n = 7	Kendall’s tau	***0*.*715***	0.073	-0.073	0.095	0.429
Later Neandertals	Spearman’s rho	***0*.*764***	-0.132	-0.046	0.422	**0.513**
open: n = 5, mixed: n = 11, wooded: n = 5	Kendall’s tau	***0*.*650***	-0.103	-0.044	0.303	**0.425**
Upper Paleolithic Modern Humans^a^	Spearman’s rho	-0.438	0.100	0.120	-0.239	0.020
open: n = 7, mixed: n = 12	Kendall’s tau	-0.367	0.084	0.103	-0.200	0.017
Upper Paleolithic Modern Humans	Spearman’s rho	**0.554**	-0.048	-0.128	**0.471**	-0.169
Aurignacian: n = 5, Gravettian: n = 11, Magdalenian: n = 4	Kendall’s tau	**0.446**	-0.013	-0.068	**0.366**	-0.127

*Asfc*: Complexity, *epLsar*: Anisotropy, *Smc*: Scale of maximum complexity, *Tfv*: Textural fill volume, *HAsfc*: Heterogreneity.

Significant correlations with *p* < 0.05 are represented in bold, with *p*<0.01 also represented in italics.

^a^ Abri Labatut specimen was excluded from this analyses since no paleoecological data is available from this site.

**Table 2:** Comparisons among the Neandertal groups from different paleoecological categories.

a) Central tendencies	*F*	*df*	*p*
MANOVA Wilks’ λ	2.333	25	**0.002**
ANOVA *Asfc* (Complexity)	8.338	5	**0.000**
ANOVA *epLsar* (Anisotropy)	0.548	5	0.738
ANOVA *Smc* (Scale of maximum complexity)	1.069	5	0.400
ANOVA *Tfv* (Textural fill volume)	1.479	5	0.231
ANOVA *HAsfc* (Heterogreneity)	1.689	5	0.173

Significant values with *p*<0.05 are represented in bold.

**Table 3:** Pairwise comparisons among the Neandertal groups from different paleoecological categories.

		Value	Value
		Open Habitats	Mixed Habitats
Early Neandertals			
Wooded Habitats	*Asfc* (Complexity)	18.21‡	11.21†
Later Neandertals			
Wooded Habitats	*Asfc* (Complexity)	20.20‡	12.78‡
Mixed Habitats	*Asfc* (Complexity)	7.42†	

Only significant differences (*p* < 0.05) for Fisher’s LSD test (†) or both Tukey’s HSD and Fisher’s LSD tests (‡) are represented.

For the Upper Paleolithic humans, unlike the case for the Neandertals, no significant correlation is detected through the paleoecological ranks (Table 1). Similarly, the MANOVA model does not detect any significant differences in microwear signatures among Upper Paleolithic humans grouped by paleoecological category (Table 4). However, significant correlation occurs between two microwear variables, complexity and textural fill volume, and archaeological complex (Table 1). Both variables increase as technological complexes change through time (Table 1). The 2-way MANOVA model detects significant differences among the groups from different technological complexes (Table 4). These differences are driven by two variables, complexity and scale of maximum complexity (Table 4). The post-hoc tests for this model show that the Magdalenians have significantly higher complexity values than the two earlier modern groups (Table 5). Moreover, the Gravettians have scale of maximum complexity values that are significantly higher than those of the Magdalenians (Table 5). This, however, is driven by one outlier in the Gravettian sample with very high scale of maximum complexity value (see [14] for details), and thus this significant difference in scale of maximum complexity among the Upper Paleolithic groups will not be discussed further. It should be noted that the two-way MANOVA results are maintained regardless of the paleoecological assignment of the Labatut 1 individual (Tables 4 and 5). Finally, the two-way MANOVA model does not detect any significant differences for the interaction between technological and paleoecological grouping indicating that microwear signatures of individuals belonging to each technological complex are similar regardless of their local environmental conditions (Table 4).

**Table 4:** Comparisons among the modern human groups from different paleoecological and technological categories: a) with Labatut 1 assigned to an open category, and b) with Labatut 1 assigned to a mixed category.

**a) Labatut 1 assigned to an open category**			
**Central tendencies**	***F***	***df***	***p***
MANOVA Wilks’ λ—Technology	2.555	10	**0.036**
ANOVA *Asfc* (Complexity)	5.473	2	**0.018**
ANOVA *epLsar* (Anisotropy)	1.599	2	0.237
ANOVA *Smc* (Scale of maximum complexity)	6.587	2	**0.010**
ANOVA *Tfv* (Textural fill volume)	1.257	2	0.315
ANOVA *HAsfc* (Heterogreneity)	2.039	2	0.167
MANOVA Wilks’ λ—Paleoecology	0.526	5	0.752
MANOVA Wilks’ λ—Technology* Paleoecology	0.703	10	0.711
**b) Labatut 1 assigned to a mixed category**			
**Central tendencies**	***F***	***df***	***p***
MANOVA Wilks’ λ—Technology	2.687	10	**0.029**
ANOVA *Asfc* (Complexity)	4.779	2	**0.026**
ANOVA *epLsar* (Anisotropy)	2.285	2	0.138
ANOVA *Smc* (Scale of maximum complexity)	6.166	2	**0.012**
ANOVA *Tfv* (Textural fill volume)	1.219	2	0.325
ANOVA *HAsfc* (Heterogreneity)	1.486	2	0.260
MANOVA Wilks’ λ—Paleoecology	0.685	5	0.645
MANOVA Wilks’ λ—Technology* Paleoecology	0.567	10	0.822

Significant values with *p*<0.05 are represented in bold.

**Table 5:** Pairwise comparisons among the modern human groups from different technological categories.

	Value^a^	Value^a^
	Aurignacian	Gravettian
Magdalenian		
*Asfc* (Complexity)	10.30‡	9.86‡
*Smc* (Scale of maximum complexity)		-8.93‡

Only significant differences (*p* < 0.05) for Fisher’s LSD test (†) or both Tukey’s HSD and Fisher’s LSD tests (‡) are represented.

^**a**^Post-hoc tests yield the same values whether Labatut 1 is assigned to an open or mixed category

The comparisons between Neandertals and earlier (i.e., Aurignacian and Gravettian) modern humans from similar paleoecological settings show that these groups are differentiated by two variables, complexity and textural fill volume (Table 6). Complexity differentiates modern humans and Neandertals from open habitats, with the former having marginally significantly higher values (Table 7). Textural fill volume, differentiates the Neandertals from mixed habitats from modern humans from both open and mixed habitats such that the Neandertals have marginally significantly higher values than the modern groups (Table 7).

**Table 6:** Comparisons between Neandertals and early modern humans.

Central tendencies^a^	*F*	*df*	*p*
MANOVA Wilks’ λ	1.900	15	**0.036**
ANOVA *Asfc* (Complexity)	2.999	3	**0.046**
ANOVA *epLsar* (Anisotropy)	0.136	3	0.938
ANOVA *Smc* (Scale of maximum complexity)	0.276	3	0.842
ANOVA *Tfv* (Textural fill volume)	3.094	3	**0.041**
ANOVA *HAsfc* (Heterogreneity)	2.127	3	0.117

Significant values with *p*<0.05 are represented in bold.

^a^ Labatut 1 was excluded from this analysis since no paleoecological data is available from this site.

**Table 7:** Pairwise comparisons between the Neandertals and modern humans from open and mixed paleoeocological categories.

		Modern Humans^a^	
		Value	Value
		Open	Mixed
Neandertals			
Open	*Asfc (Complexity)*	-14.79†	
Mixed	*Tfv (Textural fill volume)*	11.44†	9.78†

Only significant differences (*p* < 0.05) for Fisher’s LSD test (†) or both Tukey’s HSD and Fisher’s LSD tests (‡) are represented.

^a^ Labatut 1 was excluded from this analysis since no paleoecological data is available from this site.

## Discussion

**Fig 1:**
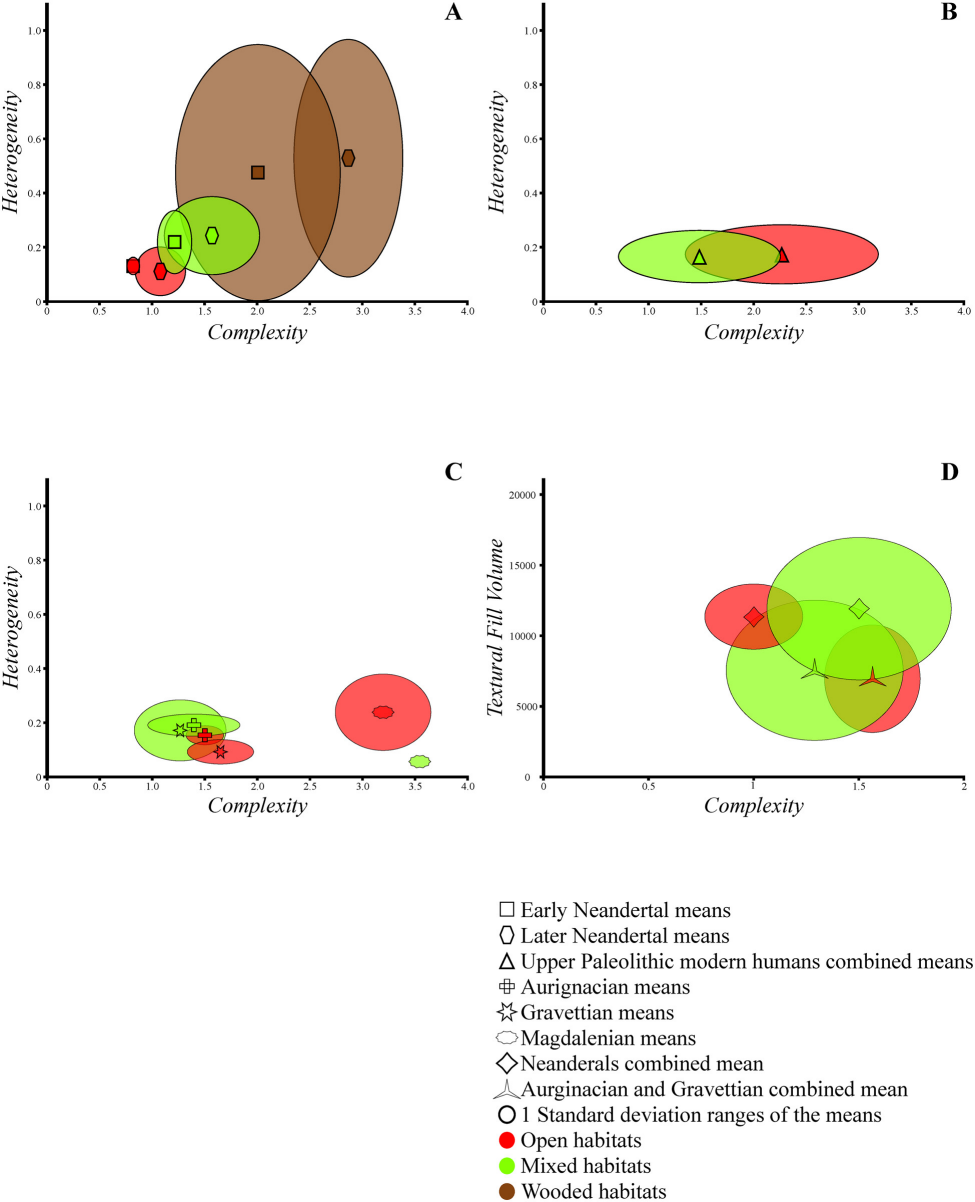
Bivariate plots of microtexture variables’ means and 1 standard deviations of Paleolithic groups. (A) Bivariate plot of complexity and heterogeneity for the early and later Neandertal specimens each grouped by paleoecological category. (B) Bivariate plot of complexity and heterogeneity for the Upper Paleolithic specimens grouped by paleoecological category. (C) Bivariate plot of complexity and heterogeneity for the Upper Paleolithic specimens grouped by both paleoecological and technological categories. (D) Bivariate plot of complexity and textural fill volume for the Neandertals (both early and later) and earlier (Aurignacian and Gravettian) modern human specimens grouped by paleoecological category.

All hominins that lived in western Eurasia during the Pleistocene, whether the Neandertals, their direct ancestors or their modern successors, had to develop strategies that allowed them to cope with changes in food supply that accompanied the multisecular fluctuations in climatic conditions. Among the Neandertals, the significant correlation between molar microwear textures and the prevailing paleoecological conditions, as represented by vegetation cover, shows that they altered their diets in response to changes in food resource availability. The microwear data suggest that both early and later Neandertals followed the same pattern of dietary alteration: as conditions became more wooded, they significantly intensified their exploitation of hard, brittle abrasive food items; whereas they evidently did the opposite as conditions became more open. Indeed, there is a significant increase in complexity in microwear with tree cover for the two Neandertal groups (Tables 2 and 3, Fig 1A). It should be noted that unlike shearing, where abrasive particles are dragged across the surface due to the direction of movement that is almost parallel to the facet surface producing higher anisotropy, crushing such particles between opposing occluding facets in a motion perpendicular to the facet plane can result in higher complexity [39]. Even though foods with abrasive mechanical properties as well as environmental abrasives have been shown to raise surface complexity values of the crushing/grinding molar facets in recent hunter-gatherers [15, 40], the level of ingestion of the former, rather than the latter, is interpreted as being responsible for driving the complexity values in early and late Neandertals. This is because the highest of these values for the fossil groups (i.e., for that from wooded habitats) remain substantially lower than what would be expected if they regularly ingested exogenous abrasives (see [11, 15–14] for detailed discussions). In addition to the changes in the level of hard foods with vegetation cover, the results of this study are consistent with a trend for increased individual dietary variability in late Neandertals from wooded areas compared to those from open ones—note the significant positive correlation between the former’s heterogeneity values and tree cover (Table 1, Fig 1A). A similar tendency appears to be present among early Neandertals as well, although this lacks statistical support (Table 1, Fig 1A).

This pattern of dietary change observed in the Neandertal lineage is perhaps not surprising considering that more wooded environments, especially Mediterranean forests, which include almost all individuals in this category (see S1 File), support a much greater diversity of potential animal and plant foods compared to the open steppe habitats that prevailed in most of Europe especially during the cold episodes of the Pleistocene. Regular consumption of different foods including a diversity of hard plant parts, such as seeds and nuts, by early and later Neandertals living in wooded areas might have increased the complexity and heterogeneity of their microwear signatures. On the other hand, the considerably lower texture complexities and heterogeneity in individuals from open environments could reflect a reduction in the amount of hard plant food items in the diet and perhaps an increased reliance on meat (which, by itself, may not cause abrasion on occlusal molar surfaces) leading to an overall narrowing of their dietary spectrum. These results are consistent with other lines of evidence that suggest a diet mostly limited to terrestrial animal protein for Neandertals living in the more open and cold environments of Europe [30, 41–47] but dietary broadening through the consumption of various plant resources, in addition to small prey and aquatic animals, for those living under temperate Mediterranean conditions in southern Europe [48–55]. Overall, the results of this study show that the changes in Neandertal diets were directly associated with changes in local environments. As such, they can be described as having been largely environmentally-driven.

However, the changes in food resources that mirrored climatic changes do not appear to have had the same effects on the diet of Upper Paleolithic individuals as they did on that of the Neandertals. In fact, the negative correlation between tree cover and the complexity of the occlusal molar surface textures, even if it does not reach a level of statistical significance (Table 1, Fig 1B), suggests a pattern of association with paleo-vegetation cover opposite of that observed for Neandertals. Yet, when considering technological association of the Upper Paleolithic individuals along with their habitat attributions, it becomes apparent that the highly complex occlusal molar surfaces of the individuals from Magdalenian contexts, almost all of which lived in open habitats, largely drive these results (Fig 1C). In comparison, the Aurignacian and Gravettian individuals, whether associated with open or mixed habitats, have significantly lower complexity values (Fig 1C). Therefore the results of this study suggest that for Upper Paleolithic humans overall there is a broad overlap of microwear signatures of individuals from open and mixed habitats within each of the technological groups but a chronological separation of these signatures, namely between the earlier (Aurignacian and Gravettian) and the later (Magdalenian) groups (Tables 4 and 5).

On the one hand, the uniformity in textures, and thus the close clustering, of Upper Paleolithic individuals from a single technological group regardless of their local paleo-habitat might suggest that, unlike earlier inhabitants of western Eurasia, the Upper Paleolithic humans within each techno-cultural unit were able to maintain dietary stability despite fluxes in their local environment. Thus, they were able to free themselves from environmental constraints probably with the aid of their technology. This, however, remains to be confirmed with microwear analyses of Upper Paleolithic individuals that lived in wooded habitats considering that, for the Neandertals, the individuals associated with tree-dominated landscapes showed the most distinct microwear signal among the different paleoecological groups examined (Table 3).

On the other hand, the clear separation in microwear patterns between the earlier and later Upper Paleolithic indicates a change in diet through time, with the most notable shift associated with the Magdalenian. The similarity in microwear signatures between the Aurignacian and Gravettian groups suggests that diet was maintained with little, if any, change during this cultural transition. It is possible that this cultural change actually aided humans in maintaining their diet by allowing them continuous access to their preferred resources at a time of deteriorating climatic conditions at the onset of the general cooling trend leading to the LGM. However, the Magdalenian cultural boom seems to have had a different effect on the human diet. The microwear data shows that this latter transition was coupled with a radical shift in diet to one that comprised substantially more hard items as is evident from the Magdalenians' significantly more complex microwear textures than those of earlier Aurignacian and Gravettian people (Fig 1C). Through the comparison of the microwear patterns of these three Upper Paleolithic groups to those of recent hunter-gatherers with known, differing diets, El Zaatari and Hublin [14] interpreted the relatively high surface complexity of crushing molar facets of the Magdalenians as resulting from an increased reliance on hard plant foods for subsistence compared to earlier periods of the Upper Paleolithic. This comparatively higher level of consumption of hard plant foods by the Magdalenians would have been expected if they were living in wooded habitats that generally offer a higher diversity of and, thus, easier access to, such food options compared to more open vegetation settings. But, even though this Late Upper Paleolithic culture developed at a time of overall warming after the LGM, it did not coincide with warm conditions in western Eurasia. At this final stage of the Paleolithic, most of the European continent was affected by some very cold episodes, such as, the Pomeranian (17–16 ka cal BP) and Older Dryas (14.2–13.7 ka cal BP) stages, which led to the accumulation of several meters of loess in Central Europe [56]. Thus, the Magdalenians had to colonize mostly open habitats, not very different from those inhabited by many of the modern populations in the region during MIS 3. Accordingly, paleoecological reconstructions associate all the Magdalenian specimens included in this study with relatively cold-open steppe biome type (S1 Table). In this kind of habitats, mechanically demanding plant food options would have been available. Underground storage organs, which would have existed in such biomes, fit this dietary category and have the potential of forming a year-round rich source of energy and nutrients [57] even though the extraction of their nutritional benefits would have come at the cost of requiring considerable work and sophisticated equipment [58–59]. The homogeneity of the microwear textures within the Magdalenian sample is consistent with the notion that the Magdalenians often selected such foods. Thus, the Magdalenians seem to have followed a different resource exploitation strategy compared to the earlier Gravettian and Aurignacian people. This change in landscape exploitation which would have been made possible by the Magdalenian’s relatively advanced and diversified toolkit might have been due, at least in part, to the overall decline in big game numbers and diversity compared to earlier periods of the Pleistocene [60].

Similarly, the present results also reveal differences in the strategies employed by earlier (i.e., Aurignacian and Gravettian) modern humans and Neandertals to exploit the western Eurasian landscape, although these differences appear to have been much more subtle compared to those that set apart the former group from the Magdalenians. In spite of the broad overlap of the microwear signatures of earlier modern humans from open and mixed habitats, when the signatures of each of these groups are compared to those of Neandertals from analogous habitats, significant differences in microwear textures are observed indicating that these two hominin taxa selected different dietary resources in comparable paleoecological settings (Table 6). Specifically, whereas Neandertals in cold-open conditions relied almost exclusively on foods that were tough rather than hard (i.e., most likely animal meat, see [11] for details), under similar habitats, the comparatively more complex crushing molar facet surfaces of the early modern humans indicate that their diet, although still predominantly comprised of tough foods, also included small amounts of hard plant foods proportional to those consumed by Neandertals from mixed habitats (Table 7, Fig 1D). However, it should be noted that the microwear textures of the early moderns that inhabited the western Eurasian open steppes still differed from those of the Neandertals from mixed habitats by having significantly lower textual fill volume (Table 7, Fig 1D). This suggests that these modern humans either selected items with generally lower hardness level or that they processed equally hard items in a way different from the Neandertals making them less mechanically demanding for consumption. Thus, the microwear data suggest that whereas Neandertals relied solely on animal meat in open habitats and only exploited plants as they became more available and diverse, modern humans seem to have indulged in plant exploitation more extensively and to have used plants to supplement their diets even in open habitats where they would have been less abundant in comparison to wooded habitats. This microwear evidence for plant exploitation and processing by Upper Paleolithic people during MIS 3 is in agreement with various botanical remains and archaeological findings of plant processing equipment from sites dating to this time period [61–64].

As for modern humans from mixed habitats, their microwear surface texture complexity values range rather widely, encompassing the majority of those of Neandertals from both open and mixed habitats (Fig 1D). This suggests a higher level of dietary variability among these modern individuals compared to Neandertals from habitats with equivalent tree cover. Yet, slight differences in diet between the earlier modern humans from mixed habitats and their Neandertal counterparts might have also been present as is indicated by the former's display of lower textual fill volume, which possibly reflects the ingestion of less mechanically demanding foods (Table 7). Overall, the differences in microwear textures between modern humans and Neandertals living in analogous environments suggest that, under similar ecological settings, the MIS 3 modern humans in Europe practiced a different pattern of food selection—perhaps also food processing—than their Neandertal predecessors. The microwear data hint that while Neandertals seem to have followed a more opportunistic dietary strategy, exploring resources only when they were most abundant and easily accessible in their local habitat (i.e., almost exclusively animal protein in open conditions but substantial amounts of plants in wooded ones), modern humans seem to have been willing to invest more effort in extracting resources from their environment (e.g., more plant foods in open conditions compared to Neandertals).

On a more general level, the results of this study show that whereas Neandertals maintained the same pattern of dietary alteration with climatic change for hundreds of thousands of years, modern humans significantly changed their dietary strategies—along with their culture at times—in a relatively much shorter time span. It is worth noting here that the microwear texture data reveal that the Neandertals continued to alter their diets in response to environmental changes in the same way that allowed them and their immediate ancestors to survive numerous climatic cycles well into MIS 3. Indeed, based on their microwear signatures, the MIS 3 Neandertals fall into the expected distinctive clusters that correspond to habitat type and do not display any evidence for significant shifts in diet that could be indicative of dietary stress brought about by the climatic fluctuations of that stage (see [11] for details). Thus, the results of this study do not support the view that the Neandertals’ disappearance was primarily due to their inability to adapt to the severe climatic fluctuations of MIS 3. But, starting at around 42 ka cal BP, modern humans came into Western Europe, having likely entered Eastern Europe a couple of millennia earlier [3]. This could have potentially brought about competition with the Neandertals making them face an extra survival pressure [65]. If, indeed, there was any competition, and if behavioral differences like the ones suggested in this study were already established at the time of first contact, these differences might have given modern humans an advantage over the Neandertals by enabling more efficient exploitation of dietary resources in their environment and more flexibility in changing the percentages of contributions of these different resources in their diets. Thus, such differences could have played a role in the demise of the Neandertals and the survival of the modern humans. Unfortunately, since most recent dating attempts and re-evaluations provide date ranges with minimum limits in the most part extending until or slightly beyond 42 ka cal BP for the youngest Neandertals included in this study [66–70], and since there is no secure evidence that, at this time period, the ranges of these specific Neandertal individuals overlapped with those of Aurignacian modern humans in Europe [3], the microwear signatures of the geochronologically youngest Neandertals included here would not reflect possible effects of direct competition with modern humans on their diet.

## Supporting Information

S1 File Additional information on the Paleolithic specimens included in this study. (DOCX)

S1 Table Microwear data for the Paleolithic specimens included in this study. (DOCX)
