# Supplementary material for: Neandertal versus Modern Human Dietary Responses to Climatic Fluctuations
Source: PLoS One. 2016 Apr 27;11(4):e0153277. doi: 10.1371/journal.pone.0153277 (PMC4847867; doi:10.1371/journal.pone.0153277)
Supplement: S1 File — (DOCX) [file pone.0153277.s001.docx]

**SI File. Additional information on the Paleolithic specimens included in this study.**

Farincourt

Farincourt 1 (RM_1_) was recovered from layer B of Farincourt Cave III. This layer is attributed to the Final Magdalenian. No absolute dates are available for this layer, but it is believed to date to the Bolling-Allerød transition of the Tradi-Wurm Interstadial, i.e., to around 12,000 BP [1-3]. Reindeer dominate the faunal assemblage from layer B, but Mammoth is also abundant. Horses and *Bos/Bison* are very rarely represented [2-3]. Thus, the faunal remains support the prevalence of cold climate with open vegetation at the time of the deposition of the hominin remains.

Saint Germain La Rivière

One tooth 1970-7-6 (RM_2_) was included in this study. This isolated tooth was found in the upper layer of the Grand Abri of Blanchard’s excavation of the site and is attributed to the Middle Magdalenian (Magdalenian III) [4]. Similar Magdalenian III layers from other parts of the site have been dated using radiocarbon dating to between 15,780 (±200) and 14,100 (±160) BP [5]. Reindeer and saiga antelope dominate the faunal assemblage from the layer of the tooth included in this study. Some remains of ibex, horse, bovids, wolf, fox, and brown bear are also represented, but in much lower quantities. This assemblage indicates open, cold-steppe conditions [4, 6].

Lachaud

The individual Lachaud 3 (RM_2_) included in this study comes from Proto/Lower Magdalenian context [7-9]. The macromammals are dominated by reindeer. Horse and saiga antelope are also represented [7-8]. The micromammals of this layer are dominated by two genera *Arvicola* (*A. amphibious* or *A. terrestris*) and *Microtus* (*M. agrestis* and *M. arvalis*) [7-8]. The former taxa are known to inhabit palearctic areas, from Great Britain to Siberia, and they prefer open wet areas like rivers, lakes, etc, whereas the latter taxa can be present in a variety of environments but prefer cold climate and open landscape [10]. Thus, the macro- and micro- faunal assemblages indicate cold and open conditions during the deposition of the layer containing Lachaud 3.

Abri Pataud

Abri Pataud 1 (LM^1^) is analysed in this study. This specimen was recovered from Level 2 of the site. This level has been attributed to the Proto-Magdalenian [11] which has been recently renamed the Final Gravettian by some authors [e.g., 12]. Radiocarbon dating place this specimen at around 22,000 BP (with calibrated dates ranging between 27,800 and 25,900 cal BP) suggesting that Abri Pataud Level 2 occupation preceded the extreme cold conditions of Heinrich event 2 [11, 13]. Pollen analyses show the presence of a mixed environment of steppe and forest in the vicinity of the Abri Pataud site at the time of deposition of Level 2 [14]. Tree refugia, areas dominated by *Pinus* and *Quercus* species, were present in the sheltered parts of the valley in front of the Abri Pataud site, whereas steppe vegetation dominated on the surrounding hills [14]. The macrofaunal assemblage from Level 2 is largely dominated by reindeer. Red deer, horses, *Bos/Bison*, Alpine ibex, and chamois are more or less represented equally in the assemblage [15]. Mammoth is also represented in lower percentages [15]. The association of species typical of cold, open habitats (i.e., reindeer) and species that prefer more temperate environments (i.e., red deer and small bovids) indicate a mixed habitat of open, steppe vegetation with wooded spaces present at least in vicinity of the site [15].

Isturitz

Isturitz 115 (LM_2_) recovered from level III of the Grande Salle of the site of Isturitz is included in this study [16-17]. The archaeological context of this level is considered Gravettian [16-18] (although Gambier [19] notes that the human remains from this level might be of Magdalenian context instead). Pollen analyses indicate that an open, cold-steppe prevailed during the accumulation of this level with arboreal pollen forming less than 5% [20].

Dolní Věstonice and Pavlov

These two sites are discussed together due to the geographical and temporal proximity of their deposits. Dolní Věstonice 13 (LM^1^), 15 (RM^1^), 16 (LM_2_), and 31 (RM_3_) and Pavlov 1 (RM^2^) specimens were included in this study. These specimens belong to the Pavlovian (Gravettian) archaeological context [21-22]. Radiocarbon dates are available for these specimens: DV13 and DV15 date to 26,640 (±110) BP [23], DV16 dates to 25,570 (±280) BP and 25,740 (±210) BP [24], DV31 dates to between 27,100 and 26,900 BP and Pavlov 1 dates to 26,170 (±450) BP [25]. Pollen analyses indicate a forest-steppe environment with coniferous and deciduous trees, as well as grasses, sedges and mugworts [26-27]. Such a reconstruction is also supported by the plant macro-remains and faunal assemblages [28-29].

Předmostí

Předmostí 21 (RM_1_) was included in this study. This specimen was recovered from Pavlovian (Gravettian) archaeological context [30]. The Gravettian layers of the site were radiocarbon dated to between 24,340 (±120) BP and 26,780 (±140) BP [30]. The plant remains from the Gravettian layers show a mix of forest-steppe environment [see 27]. Similarly, the faunal assemblage consists of a diversity of taxa including forest and open landscape species, as well as wetland and arid steppe species [27].

Abri Labatut

The Abri Labatut 1 (LM3) individual analysed in this study comes from the Upper Perigordian (Gravettian) context of Abri Labatut, Castel Merle caves [31]. Unfortunately, no paleoecological reconstructions are available for the layers of Abri Labatut.

Cro-Magnon

Cro-Magnon 2 (RM^1^) is included in this study. This specimen is attributed to the early Gravettian period [32]. A Littorina shell associated with the Cro-Magnon human remains provided a radiocarbon date of 27 680 (±270) BP [32]. The associated macrofauna is dominated by reindeer and horse, and also included mammoth. This faunal assemblage is indicate cold, open conditions [33].

Barma Grande

Barma Grande 1 (LM_2_) and 2 (RM_2_) are included in this study. These individuals are of Gravettian context [34]. Barma Grande individual 2 is part of a triple burial (also including Barma Grande individuals 3 and 4). This burial was dated by archaeological association to around 25 ka [35]. Direct radiocarbon dating is available for Barma Grande individual 6. This date of 24800 (±800) BP for this individual is most likely also the date for the rest of the individuals from the site [34]. Paleoecological reconstructions are unavailable from the site of Barma Grande itself. But, pollen analyses from other Italian sites (i.e., Lago Grande di Monticchio, Lagaccione, Vico, and Valle di Castiglione) show that during that time the area was covered by a mix of open and forested vegetation [36-37]. Similar conditions were also detected in the deep sea cores from the Mediterranean [38].

Les Rois

Les Rois R50 #31 (RM_2_) is one of the isolated teeth found at the site of Les Rois and included in this study. The isolated teeth are found in Aurignacian context [39] and are dated to between 27-31 kyr [40]. The faunal assemblage from Unit 2 or layer A2 beta (i.e., the same unit/layer as the isolated tooth examined here) is dominated by reindeer [39, 41]. Horses are also present, but in much lower percentages, and Bos/Bison remains are sparse [39, 41]. This faunal assemblage is indicative of cold open vegetation.

Mladeč

Mladeč 1 (RM^1^), 2 (LM^1^), and 8 (LM^2^) are included in this study. These individuals were recovered from middle or late Aurignacian contexts [e.g., 42] and are dated to around 31 kyr by AMS radiocarbon dating [43]. A variety of species is represented in the faunal assemblage associated with the human remains. The faunal assemblage is dominated by bovids, but reindeer, horse, and mammoth are also represented [44]. The fauna indicate one of the temperate oscillations of the Interpleniglacial when the area was covered by a mix of forest and steppe vegetation [45].

Abri Blanchard

Abri Blanchard 1 (RM_3_) is included in this study. This is an isolated tooth that was recovered from the Middle Aurignacian layers [46]. Even though no paleoclimatic data is available from the site of Blanchard itself, climatic reconstructions are available from the Aurignacian layers of Abri Castanet which is very close to Abri Blanchard. Charcoal, pollen, and faunal analyses from Abri Castanet support the prevalence of cold, open vegetation at the time of the deposition of the layer containing Abri Blanchard 1 [47].

Grotta Breuil

Grotta Breuil 2 (LM_1_) Neandertal tooth was included in this study. This tooth was recovered from within the surface levels of the stratigraphic layers inside the cave [48]. Electron spin resonance dating on mammal teeth from the layers yielding the human remains provide a date of around 36.6 ±2.7 kyr [49]. The analysis of bird remains from the site suggest temperate conditions and indicates that the plain in front of the cave was rich in trees and water and that species of open land were limited in number [50]. The micromammal assemblage also indicate that temperate climate and forested vegetation prevailed at the time of deposition of the human remains. This assemblage is dominated by *Apodemus sylvaticus*, an inhabitant of wooded areas, and also includes high percentages of arboreal species, such as *Myoxus glis*, *Eliomys quercinus*, and *Dryomys nitedula* [51]. The macromammal remains dominated by *Cervus elaphus*, support such a reconstruction as well [52].

Spy

The Spy I (RM^2^) specimen included in this study is radiocarbon dated to around 36 kyr BP [53]. The associated faunal assemblage is dominated by remains of horses and reindeer and thus indicates that Spy I Neandertal lived in open vegetation prevailing during cold conditions [54]. Such a reconstruction is also supported by northern European pollen spectra which show that, open vegetation -fluctuating between tundra/shrub-tundra and steppe-tundra/temperate grassland in response to the climatic fluctuations of MIS 3- prevailed in the area of the Spy site for the entire duration of MIS 3 [55-58].

Zafarraya

The Zafarraya mandible (RM_1_) is included in this study. This mandible was recovered from the archaeological unit UE. Uranium-series dates on animal bone and teeth found in association with the mandible provide an age of 33.4 ±2 kyr for this specimen [59]. Various lines of data, including sedimentological, palynological, paleontological (i.e., macromammals, micromammals, birds, reptiles, amphibians), support a reconstruction of the landscape in the area similar to today’s with the presence of different ecological niches and with the forest character evident by the prevalence of various woodland loving taxa [60]. For example, the remains of *Cervus elaphus* are the second most dominant in the faunal assemblage (*Capra pyrenaica* remains are the most dominant) whereas animal species generally associated with open vegetation (i.e., *Equus*) are found in low percentages [60]. Among the trees, oak is the most dominant [60-61].

St. Césaire

St. Césaire 1 (RM^1^) was included in this study. This individual was recovered from EJOP superior level [62]. Sedimentary analysis points to the beginning of a cooling trend in this level and describe the climatic conditions prevailing at the time of deposition of this level as transitional between the earlier warmer conditions associated with the lower levels and the colder ones that followed [62]. Such a transitional climatic character is supported by the microfaunal remains which show an increase in the percentages of the narrow-skulled vole and the decrease in those of common vole compared to the lower levels of the site [63-64]. The macrofaunal remains recovered from the EJOP level also reflect intermediate climatic conditions since they include a mix of species indicative of cold as well as temperate climatic conditions [65-67]. Although these remains are dominated by taxa such as reindeer, steppe bison, and horse, and also include woolly rhinoceros and mammoth and thus indicate the prevalence of open vegetation, the existence of forest-dwelling species such as boar, red deer, and megaceros, indicate the presence of wooded vegetation in the vicinity of the site [65, 67]. Moreover, three pollen spectra are available from the EJOP level of Saint-Césaire. These spectra differ in the frequencies of arboreal pollen (from 80% to 50% to 20%) mostly as a result of differences in frequencies of pine pollen [68]. Even if the spectrum with the highest frequency of arboreal pollen most likely represents an overestimation due to the high frequency of pine pollen in the part of the site the samples were collected from [68], the pollen data does confirm the presence of trees (dominated by pine, followed by juniper) in the vicinity of the cave indicating that the conditions were not completely open. The combination of the different lines of data allows for safely reconstructing the vegetation of the EJOP superior level as a mixture of both open and wooded elements with the latter consisting mostly of coniferous taxa.

Vindija

Vindija Neandertal specimens 11.45 (LM_2_), 11.46 (LM^1^), and 12.1 (RM^2^) are included in this study. These specimens were recovered from layer G3 [69] which has been dated to at least 42 kyr based on amino acid racemization and U/Th methods [70]. The microfaunal species from this layer which include *Arvicola terrestris, Microtus ex gr. arvalis/agrestis, M. oeconomus, Microtus ex gr. subterraneus/multiplex, Chionomys glareolus,* and *Marmota marmot*s are indicators of a temperate, continental climate with a generally open biotope characterized by some forest cover less than what is present in the region today [71]. The macrofaunal assemblage which includes remains of *Stephanorhinus sp., Cervus elaphus, Alces alces, Megaloceros giganteus, Capreolus capreolus, Bos primigenius, Capra ibex, and Rupicapra rupicapra*, is in agreement with the climatic/vegetation reconstruction provided by the micromammal remains and also indicate broadly temperate conditions with a range of environments (open, forested, wetland, and rocky) present in the region [71].

Lakonis

Lakonis LKH1 (LM_3_) was included in this study. This is an isolated Neandertal tooth that was recovered from the Initial Upper Paleolithic layer and dated to between 44-38 ka [72]. The site of Lakonis is located in a coastal area in southern Europe that provided refugia for trees and maintained a constant vegetation cover during the climatic fluctuations of MIS 3 [58]. The faunal assemblage from the site suggests the presence of diverse ecological setting around the cave with the presence of dense woodlands in addition to some parkland vegetation [73]. Analyses of charcoal samples, although inconclusive, indicate the presence of trees belonging to the genus *Prunus* [73].

El Sidrón

El Sidrón Adult 1 (LM_1_) was included in this study. The Neandertals from El Sidrón were recovered from Unit III of the cave’s Galería del Osario [74]. These remains are dated to around 49,000 BP [75]. The analyses of the faunal assemblage and anthracological samples associated with the hominin remains show that, during the deposition of Unit III, climatic conditions were warm and humid and similar to the present with a generally wooded landscape covered with a mix of coniferous and deciduous forests in addition to few open spaces [76-80].

Petit-Puymoyen

Petit-Puymoyen 2 (LM^1^) and 4 (RM_1_) are included in this study. Analyses of macrofaunal remains associated with the hominins provide information on the environmental conditions prevailing at the time of deposition of the Petit-Puymoyen Neandertals sampled in this study. These analyses indicate a temperate climate that was not extreme in either way and implies mixed vegetation which was not entirely open nor entirely forested [81].

Amud

Amud 1 (RM_2_) individual was included in this study. This individual was recovered from layer B1 which was dated to around 55 ka by TL dating (on burnt flint) [82] and to around 53 ka by ESR and Th/U dating (on associated faunal teeth) [83]. The macrofaunal assemblage from the same layer as Amud 1 is dominated by remains of *Gazella*, followed by *Dama* [84]. The dominance of these taxa, along with the presence of remains of *Cervus*, *Bos*, and *Sus*, in the assemblage reflect the existence of wooded vegetation in the area close to the cave [84-85]. A recent analysis of the micromammalian assemblages from Amud Cave also support the presence of a Mediterranean woodland habitat in the vicinity of the site during the time of the Neandertal occupation [86]. It should be noted that the documented abundance of the open habitat dweller, *Microtus guentheri*, remains in the micromammals assemblages reflects a taphonomic effect caused by the selective accumulation of this taxa by *Tyto alba* rather than an indication of open habitats [86]. Even though regional pollen data reconstruct the vegetation of the Levant during MIS 3 as generally open with steppe or semi-desert elements dominating over trees, during several warm episodes this open vegetation was replaced by severe park-forest episodes with coniferous and deciduous trees dominating [58, 87]. One of these episodes has been dated to between 58-54 ka [58, 88-89] and thus broadly coincides with the available dates of Amud I Neandertal.

Kalamakia

Kalamakia KAL 3 (LM^3^) specimen is included in this study. This is an isolated Neandertal tooth which comes from the upper part of Unit III of the site [90]. This Unit is dated to the Middle Paleolithic, between 100 and >39 ka [90]. Palynological data show that during this time period the area was covered with a mix of Mediterranean shrubland and pre-steppic forest taxa [90-91]. The macro- and micro-faunal assemblages which consist of species that generally prefer mixed and open forest vegetation support this reconstruction [90, 92].

Kebara

Kebara 2 (LM_2_) was included in this study. This individual was recovered from layer XII and is dated by TL dating to around 59 kyr [93] and by ESR dating to between 64 and 60 kyr [94]. The macrofaunal assemblage from the Mousterian layers of the site of Kebara show the dominance of two species, *Gazella gazella* and *Dama mesopotamic*, along with the presence of other taxa, including *Bos primigenius*, *Cervus elaphus*, *Sus scrofa,* and *Equus sp.*, albeit in lower frequencies [95-98]. The micromammal assemblage is indicative of both wooded and open habitats in the vicinity of the cave. Aside from overwhelming majority of *Microtus guentheri* remains (which might be a taphonomic effect), open (*Meriones tristrami*) as well as wooded (*Apodemus spp.*) habitat microfaunal taxa are represented in equal frequencies [99-100]. Thus, overall, the faunal data indicate that the vegetation of the area was a mix of woodland and semi-desert at the time of deposition of the layer containing Kebara 2.

La Quina

Dental remains representing two Neandertal individuals, 5 (RM_2_) and 20 (RM^2^), from the site of La Quina are included in this study. These individuals were recovered from Layer 3 of Trench B (La Quina 5) and Layer 2 of Trench C (La Quina 20) of L Henri-Martin La Quina Amont stratigraphy and most likely correlate respectively with layers L through Q and D through K of the lower (i.e., inferior or south) deposits of the Debénath and Jelinek’s stratigraphy [101-102]. No absolute dates for the layers of the specimens are available, but based on absolute dates available for the higher layers of the sequence and on relative dating based on archaeological correlation of the layers containing the Neandertal individuals included in this study, La Quina 5 is placed in either MIS 3 or 4 and La Quina 20 in MIS 4 [103]. The faunal assemblages associated with both Neandertal individuals studied are dominated by remains of reindeer and horse suggesting the prevalence of open habitat [104-106].

Subalyuk

Subalyuk 1 (LM_2_) is included in this study. This specimen is believed to have come from Layer 11 and is dated based on microfaunal association to between 70-60 kyr [107]. The associated microfaunal assemblage which includes *Ochotona pusillus*, *Allactaga saliens*, and the macrofaunal assemblage which records strong presence of *Rangifer tarandus* are greatly indicative of continental steppe conditions [107-108].

Grotte de l'Hyène (Arcy-sur-Cure)

Grotte de l'Hyène IVb6 B9 (RM_1_) was included in this study. This specimen was recovered from level 20 [109]. Based on correlations with other sites, this layer is dated to the cold period between the Brörup and Odderade interstadials and thus equated with OIS 5b [109]. Sedimentological analysis indicates that the deposits the Neandertal remains were found in represent a cold episode reflected in cold steppe conditions [109]. The faunal taxa include *Rangifer tarandus*, *Mammuthus primigenius*, and *Rhinocerus tichorhinus* and thus also support this reconstruction [110].

Rochelot

Rochelot 1098 (LM_2_) was included in this study. This individual is dated by biostratigraphic correlation to the end of MIS 5 [111]. The faunal taxa present include boar, aurochs, wild ass, fallow deer, red deer, roe deer, giant deer, narrow-nosed rhinocerous, beaver [112]. Most of these taxa prefer sheltered wooded areas and temperate, humid climate, although the sporadic occurrence of reindeer imply that cooler climatic conditions occasionally prevailed and the abundant remains of horse do indicate the presence of open grassland in the area of the site as well [112]. Thus, the fauna associated with the Neandertal remains support mixed vegetation cover at that time [112].

Tabun

Tabun II (LM_2_) was included in this study. It was recovered from layer C which has been dated by ESR to between 120 ±16 kyr and 140 ±21 kyr [113] and by TL on burnt flint to 165 ± 16 kyr [114]. The microfauna from layer C indicates mixed vegetation elements with a rather dense bush-forest cover - as indicated by the considerable number of at least two species of *Apodemus* - which was interrupted by grasses and lower vegetation of Mediterranean character - as indicated by the preponderance of *Microtus* and the presence of the fossorial *Spalax* [115]. Unfortunately, the large mammal assemblages available from layer C cannot be used for ecological reconstructions since these most likely contain a mix of material from layers C, D, and B [115].

La Chaise - Bourgeois-Delaunay

La Chaise BD8 (LM^1^) specimen was included in this study. This specimen was recovered from (or immediately below) the base of layer 11 [116]. Absolute dates show that this layer was deposited in two fast depositional events, one dating to MIS 6 and another dating to MIS 5e, separated by a hiatus coinciding with the coldest part of MIS 6 when the cave entrance seems to have been closed [116-117]. Pollen analysis from the lower part of layer 11 shows that the vegetation was mostly open, but that trees (with *Pinus* and *Corylus* dominating) were also represented although in somewhat low percentage [118].

Montmaurin – La Niche

The mandible from this site is included in this study. Microwear data was recovered from the RM_1_. The mandible has been initially assigned to “Pre-Neandertals” [119-121] and has been dated to the end of the Riss Glaciation, around 130 kyr [113]. Pollen analyses from the same layer as the mandible indicate that the vegetation was relatively open-steppic, but that it also included a relatively low percentage of trees between 20-30% dominated by *Pinus*, with deciduous trees namely *Betula*, *Quercus* and *Corylus* also present [122-123].

Biache-Saint-Vaast

Biache-Saint-Vaast individual number 1 (LM^1^) is included in this study. Bed II A where this fossil comes from is dated to the end of MIS 7 and the beginning of MIS 6 [124]. Available absolute dates are 175 ±13 kyr (TL on burnt flints) and around 272 kyr (ESR on the enamel of dental remains) [125-126]. Evidence from vertebrates, molluscs, and pollen remains suggests a cool temperate phase between the preceding warmer interglacial and the cold glacial that followed. The vegetation was a mix of steppic and coniferous and deciduous wooded elements [124, 127-128].

Pontnewydd

Pontnewydd PN1 (LM^2^) tooth is included in this study. This tooth was recovered from the Intermediate Complex of the site which is dated to MIS 7 (129). The faunal remains recovered from the same deposits as this tooth, with species like the roe deer, beavers, and wood mouse, are indicative of wooded vegetation prevailing most likely during MIS 7c (130-131).

Steinheim

Microwear data was collected from the RM^1^ of the Steinheim skull. Based on mammalian biostratigraphy, the skull is dated to either MIS 9 or 11 (132). The faunal assemblage from the same layer that yielded the hominin skull (i.e., the Waldelefanten-Schotter Layer) consists of species like *Sus scrofa, Cervus elaphus, Bos/Bison* that represent temperate conditions (132-133). No cold adapted fauna are represented in this layer (132-133). This data shows that wooded habitats were prevalent during that time (132-133).

Atapuerca – Sima de los Huesos

Microwear data was collected from five individuals from Atapuerca – Sima de los Huesos site. These individuals are: 4 (AT-970, RM^1^), 7 (AT-270, LM^2^), 8 (AT-3177, LM^1^), 17 (AT-20, RM^1^), 19 (AT-576, LM_1_). These remains are dated to around 430 kyr using a variety of techniques (134). Palynological samples taken from the matrix of the human fossils are composed by about 40% pollen of trees of which pine is the most abundant, with deciduous oak, and evergreen oak, birch and *Fagus* also represented in decreasing order of abundance (135). A more recent analysis of faunal remains from Sima de los Huesos indicates that the hominins were living in a highly productive ecosystem that is best described as open woodland (136).

Arago

Arago 13 (RM_1_) and 54 (RM^1^) are included in this study. These specimens represent the most primitive end of our sample, sometimes assigned to “*Homo heidelbergensis*”. They do however display some incipient Neandertal features (137-138). They were recovered from Unit III of the Middle Stratigraphic Complex. This Unit pre-dates MIS 9 and could be as old as MIS 12 (139-142). The micromammals recovered from Unit III, which include arctic and Siberian taxa (e.g. *Dicrostonyx torquatus*, *Microtus (Stenocranius) gregalis*), point to cold, dry and open conditions (140). The macromammals also indicate similar conditions (143-144).

1. David S. La fin du Paléolithique supérieur en Franche-Comté: environnement, cultures, chronologie. Gallia Préhistoire. 1996;38: 111-248.

2. Joffroy R, Mouton AP. La station magdalénienne de Farincourt (H.-M.). Bull Soc Préhist Fr. 1946;43: 91-100.

3. Joffroy R, Mouton AP. Précisions nouvelles sur les stations magdaléniennes de Farincourt (Haute-Marne). Revue archéologique de l'Est et du Centre-Est. 1956;7: 193-223.

4. Blanchard R, Peyrony D, Vallois HV. Le gisement et le squelette de Saint-Germain-la-Rivière. Archives de l'Institut de Paléontologie Humaine. 1972;34.

5. Gambier D, Valladas H, Tisnérat-Laborde N, Arnold M, Bresson F. Datation de vestiges humains présumés du Paléolithique supérieur par la méthode du Carbone 14 en spectrométrie de masse par accélérateur. Paléo. 2000;12: 201-212.

6. Vanhaeren M, d’Errico F. Grave goods from the Saint-Germain-la-Rivière burial: Evidence for social inequality in the Upper Palaeolithic. J Anthropol Archaeol. 2005;24: 117-134.

7. Cheynier A. Stratigraphie de l'abri Lachaud et les cultures des bords abattus. Archivo de Prehistoria Levantina. 1953;4: 25-55.

8. Cheynier A. L'abri Lachaud à Terrasson (Dordogne). Paris: Presses Universitaires de France. 1965.

9. Ferembach D. Les restes humains de l'Abri Lachaud. Bulletin et Mémoires de la Société d'anthropologie de Paris. 1957;8: 61-80.

10. Mitchell-Jones AJ, Amori G, Bogdanowicz W, Krystufek B, Reijnders PJH, Spitzenberger F, et al. The atlas of European mammals. London: T & AD Poyser Ltd. 1999.

11. Movius HLJ. Summary of the stratigraphic sequence. In: Movius HLJ, editor. Excavation of the Abri Pataud Les Eyzies (Dordogne). Cambridge: Peabody Museum of Archaeology and Ethnology, Harvard University; 1975. pp. 7-18.

12. Djindjian F, Koslowski J, Otte M. Le Paléolithique Supérieur en Europe. Armand Colin, Paris. 1999.

13. Chiotti L, Nespoulet R, Henry-Gambier D. Occupations and status of the Abri Pataud (Dordogne, France) during the Final Gravettian. Quatern Int. 2015;359: 406-422.

14. Donner JJ. Pollen composition of the Abri Pataud sediments. In: Movius HLJ, editor. Excavation of the abri Pataud, les Eyzies, Dordogne. Cambridge: Peabody Museum of Archaeology and Ethnology, Harvard University; 1975. pp. 160-174.

15. Crépin, L. Données archéozoologiques des grands mammifères. In: Nespoulet R, Chiotti L, Henry-Gambier D, editors. Le Gravettien Final de l’Abri Pataud (Dordogne, France): Fouilles et études 2005-2009. Oxford: BAR International Series 2458; 2013. pp. 63-88.

16. de Saint Périer R. La grotte d'Isturitz, I: le Magdalénien de la salle Saint-Martin. Paris: Archives de l’Institut de Paléontologie Humaine 17. 1936.

17. de Saint Périer R, de Saint Périer S. La grotte d'Isturitz, III: Les Solutrérns, les Aurignaciens et les Moustériens. Paris: Archives de l’Institut de Paléontologie Humaine 25. 1952.

18. de Saint Périer R. La grotte d'Isturitz, I: le Magdalénien de la salle Saint-Martin. Paris: Archives de l’Institut de Paléontologie Humaine 7. 1930.

19. Gambier D. Les vestiges humains du gisement d'Isturitz (Pyrénées-Atlandiques): étude anthropologique et analyse des traces d'action humaine intentionnelle. Antiquités Nationales. 1990-1991;22-23: 9-26.

20. Leroi-Gourhan A. Résultats de l'analyse pollinique de la grotte d'Isturitz. Bulletin de la Société Préhistorique de France 1959;56: 619-624.

21. Klima B. Zur problematik des Aurignacian und Gravettian in Mittel-Europa. Archaeologia Austriaca. 1959;26: 35-51.

22. Svoboda J. The archaeological framework. In: Trinkaus E, Svoboda J, editors. Early Modern Human Evolution in Central Europe: The People of Dolní Věstonice and Pavlov. New York: Oxford University Press; 2006. pp. 6-8.

23. Klima B. A triple burial from the Upper Paleolithic of Dolni Vestonice, Czechoslovakia. J Hum Evol. 1988;16: 831-835.

24. Svoboda J, van der Plicht J, Kuželka V. Upper Palaeolithic and Mesolithic human fossils from Moravia and Bohemia (Czech Republic): some new 14C dates. Antiquity. 2002;76: 957-962.

25. Svoboda J. The archaeological context of the human remains. In: Trinkaus E, Svoboda J, editors. Early Modern Human Evolution in Central Europe: The People of Dolní Věstonice and Pavlov. New York: Oxford University Press; 2006. pp. 9-14.

26. Svobodová H. Pollen analysis of Upper Palaeolithic triple burial at Dolni Vestonice. Archeologické rozhledy. 1991;43: 505-510.

27. Musil R. Palaeoenvironment at Gravettian sites in central Europe with emphasis on Moravia (Czech Republic). Quartär. 2010;57: 95-123.

28. Manson SLR, Hather JG, Hillman GC. Preliminary investigation of the plant macroremains from Dolni Vestonice II and its implications for the role of plant foods in Paleolithic and Mesolithic Europe. Antiquity. 1994;68: 48-57.

29. West D. Analysis of the fauna recovered from the 1986/1987 excavations at Dolni Vestonice II, Western Slope. Památky Archeologické. 2001;92: 98-123.

30. Svoboda JA. The Upper Paleolithic burial area at Prĕdmostí: ritual and taphonomy. J Hum Evol. 2008;54: 15-33.

31. de Sonneville-Bordes D. Le Paléolithique supérieur en Périgord. Bordeaux: Delmas; 1960.

32. Henry-Gambier D. Les fossiles de Cro-Magnon (Les Eyzies-de-Tayac, Dordogne): Nouvelles données sur leur position chronologique et leur attribution culturelle. Bull Mem Soc Anthropol Paris. 2002;14: 89-112.

33. Lartet E. Remarques sur la faune de Cro-Magnon, d’après les débris osseux découverts soit dans la sépulture humaine, soit dans les restes de foyers placés à proximité. Revue des Sociétés Savantes. 1868;3: 296-300.

34. Formicola V, Pettitt PB, Del Lucchese A. A Direct AMS Radiocarbon Date on the Barma Grande 6 Upper Paleolithic Skeleton. Curr Anthropol. 2004;45: 114-118.

35. Schumann B. Biological evolution and population change in the European Upper Palaeolithic: University of Cambridge; 1995.

36. Follieri M, Giardini M, Magri D, Sadori L. Palynostratigraphy of the last glacial period in the volcanic region of Central Italy. Quat Int. 1998;47: 317-335.

37. Watts WA, Allen JRM, Huntley B. Vegetation history and palaeoclimate of the last glacial period at Lago Grande di Monticchio southern Italy. Quat Sci Rev. 1996;15: 133-153.

38. Genty D, Combourieu-Nebout N, Hatté C, Blamart D, Ghaleb B, Isabello L. Rapid climatic changes of the last 90 kyr recorded on the European continent. C R Geoscience. 2005;337: 970-982.

39. Vallois HV. Les restes humains d'âge aurignacien de la grotte des Rois, Charente. Bull Mem Soc Anthropol Paris. 1958;9: 138-159.

40. Michel A, d'Errico F, Lenoble A, Vanhaeren M, Ramirez Rozzi F, Grootes P, et al. Nouvelles fouilles sur le site aurignacien Chez les Rois (Mouthiers-sur-Boeme, Charente). In: Jaubert J, Bordes J-G, Ortega I, editors. Les sociétés du paléolithique dans un Grand Sud-Ouest de la France: nouveaux gisements, nouveaux résultats, nouvelles methods. Paris: Mémoire XLVII de la Société Préhistorique Française; 2008. pp. 289-299.

41. Bouchud, J. Étude partielle de la faune des Rois. In: Mouton P, Joffroy R, editors. Le gisement aurignacien des Rois à Mouthiers (Charente). Paris: Centre National de la Recherche Scientifique; 1958. pp. 100-103.

42. Szombathy J. Die diluvialen Menschenreste aus der Fürst-Johannes-Höhle be Lautsch in Mähren. Eiszeit. 1925;2: 1-34.

43. Wild EM, Teschler-Nicola MT, Kutschera W, Steier P, Trinkaus E, Wanek W. Direct dating of Early Upper Palaeolithic human remains from Mladeč. Nature. 2005;435: 332-335.

44. Pacher M. Large mammal remains from the Mladeč Caves and their contribution to site formation processes. In: Teschler-Nicola M, editor. Early Modern Humans at the Moravian Gate: The Mladeč Caves and their Remains. Wien: Springer-Verlag; 2006. pp. 99-148.

45. Svoboda J. Mladeč and other caves in the middle Danube region: early modern humans, late Neandertals, and projectiles. In: Zilhão J, Aubry T, Carvalho F, editors. Les premiers hommes modernes de la Péninsule Ibérique: Actes du colloque de la Commission VIII de l'UISPP. Lisbon: Instituto Portuguès de Arqueologia; 2001. pp. 45-60.

46. Ferembach D. Note sur la dent Aurignacienne trouvée dans l'Abri Blanchard. Bull Mem Soc Anthropol Paris. 1958;9: 199-202.

47. Pelegrin J, O'Farell M. Les lamelles retouchées ou utilisées de Castanet. In: Le Brun-Ricalens F, editor. Productions lamellaires attribuées à l'Aurignacien: chaînes opératoires et perspectives technoculturelles. Luxembourg: Musée National d'Histoire et d'Art; 2005. pp. 103-121.

48. Manzi G, Passarello P. At the archaic/modern boundary of the Genus Homo: the Neandertals from Grotta Breuil. Curr Anthropol. 1995;36: 355-366.

49. Schwarcz HP, Buhay W, Grün R. Absolute dating of sites in coastal Lazio. Quaternaria Nova. 1990-1991;I: 51-67.

50. Recchi A. Bird remains from the Upper Pleistocene sites of Grotta Breuil (M. Circeo, Latina, Italy) and Riparo Salvini (Terracina, Latina, Italy). Quaternaria Nova. 1995;V: 81-98.

51. Kotsakis T. Late Pleistocene fossil microvertebrates of Grotta Breuil (Monte Circeo, central Italy). Quaternaria Nova. 1990-1991;I: 325-332.

52. Stiner MC. Honor Among Thieves. Princeton: Princeton University Press; 1994.

53. Semal P, Rougier H, Crevecoeur I, Jungels C, Flas D, Hauzeur A, et al. New data on the late Neandertals: Direct dating of the Belgian Spy fossils. Am J Phys Anthropol. 2009;138: 421-428.

54. Cordy J. Apport de la paléozoologie à la paléoécologie et à la chronostratigraphie en Europe du nord-occidental. L'Homme de Neanderthal. Liege: Etudes et Recherches Archeologiques de l'Universite de Liege; 1988. pp. 55-64.

55. Alfano MJ, Barron EJ, Pollard D, Huntley B, Allen JRM. Comparison of climate model results with European vegetation and permafrost during oxygen isotope stage three. Quatern Res. 2003;59: 97-107.

56. Huntley B, Alfano MJ, Allen JRM, Pollard D, Tzedakis PC, de Beaulieu J-L, et al. European vegetation during Marine Oxygen Isotope Stage-3. Quatern Res. 2003;59: 195-212.

57. Huntley B, Allen JRM. Glacial Environments III: palaeo-vegetation Patterns in Last Glacial Europe. In: van Andel TH, Davies W, editors. Neanderthals and Modern Humans in the European Landscape during the Last Glaciation: Archaeological results of the Stage 3 Project. Cambridge: McDonald Institute for Archaeological Research; 2003. pp. 79-102.

58. van Andel T, Tzedakis PC. Paleolithic landscapes of Europe and environs 150,000-25,000 years ago: an overview. Quatern Sci Rev. 1996;15: 481-500.

59. Hublin J-J, Ruiz CB, Lara PM, Fontugne M, Reyss J-L. The Mousterian site of Zafarraya (Andalucia, Spain): dating and implications on the Palaeolithic peopling processes of Western Europe. Comptes Rendus de l'Academie des Sciences Serie II - Fascicule A - Sciences de la Terre et des Planetes. 1995;321: 931-937.

60. Barroso Ruiz C, Marchi F, Abdessadok S, Bailón S, Desclaux E, Gregoire S. Contexte paléoécologique, paléoclimatique et paléogéographique des Néandertaliens de la grotte du Boquete de Zafarraya. In: Barroso Ruiz C, de Lumley H, editors. La grotte du Boquete de Zafarraya (Málaga, Andalousie). Sevilla: Junta de Andalucía, Consejería de Cultura; 2006. pp. 1127-1166.

61. Barroso Ruiz C, Medina Lara P, Sanchidrian Torti JL, Ruiz Bustos A, Garcia Sanchez M. Le gisement Mousterien de la Grotte du Boquete de Zafarraya (Alcaucin, Andallousie). L'Anthropologie. 1984;88: 133-134.

62. Miskovsky JC, Lévêque F. The sediments and stratigraphy of Saint-Césaire: contributions to the paleoclimatology of the site. In: Lévêque F, Backer AM, Guilbaud M, editors. Context of a Late Neandertal: Implications of Multidisciplinary Research for the Transition to Upper Paleolithic Adaptations at Saint-Césaire, Charente-Maritime, France. Madison: Prehistory Press; 1993. pp. 7-14.

63. Marquet J-C. L’Homme de Néandertal et son environnement dans la moitié ouest de la France d’après les rongeurs. In: Otte M, editor. L’Homme de Néandertal, vol 2: L’Environnement. Liège: ERAUL; 1988. pp. 105-110.

64. Marquet J-C. Paléoenvironnement et Chronologie des Sites du Domaine Atlantique Français d'Âge Pléistocène Moyen et Supérieur d'Après l'Étude des Rongeurs. Tours: Les Cahiers de la Claise; 1993.

65. Lavaud-Girard F. Macrofauna from the Castelperronian levels at Saint-Césaire, Charente-Maritime. In: Lévêque F, Backer AM, Guilbaud M, editors. Context of a Late Neandertal: Implications of Multidisciplinary Research for the Transition to Upper Paleolithic Adaptations at Saint-Césaire, Charente-Maritime, France. Madison: Prehistory Press; 1993. pp. 71-77.

66. Patou-Mathis M. Taphonomic and palaeoethnographic study of the fauna associated with the Neandertal of Saint-Césaire. In: Lévêque F, Backer AM, Guilbaud M, editors. Context of a Late Neandertal. Madison: Prehistory Press; 1993. pp. 79-102.

67. Morin E. Late Pleistocene population interaction in western Europe and modern human origins: new insights based on the faunal remains from Saint-Césaire, southwestern France. Ph.D. Dissertation: The University of Michigan; 2004.

68. Leroyer C, Leroi-Gourhan A. Pollen analysis at Saint-Césaire. In: Lévêque F, Backer AM, Guilbaud M, editors. Context of a Late Neandertal. Madison: Prehistory Press. pp. 61-70.

69. Wolpoff MH, Smith FH, Malez M, Radovčić J, Rukavina D. Upper Pleistocene Human Remains from Vindija Cave, Croatia, Yugoslavia. Am J Phys Anthropol. 1981;54: 499-545.

70. Wild EM, Paunovic M, Rabeder G, Steffan I, Steier P. Age determination of fossil bones from the Vindija Neanderthal site in Croatia. Radiocarbon. 2001;43: 1021-1028.

71. Miracle PT, Lenardić JM, Brajković D. Last glacial climates, ‘‘Refugia’’, and faunal change in Southeastern Europe: Mammalian assemblages from Veternica, Velika pećina, and Vindija caves (Croatia). Quatern Int. 2010;212: 137-148.

72. Harvati K, Panagopoulou E, Karkanas P. First Neanderthal Remains from Greece: the Evidence from Lakonis. J Hum Evol. 2003;45: 465-473.

73. Panagopoulou E, Karkanas P, Tsartsidou G, Kotjabopoulou E, Harvati K, Ntinou M. Late Pleistocene archaeological and fossil human evidence from Lakonis Cave, Southern Greece. J Field Archaeol. 2002-2004;29: 323-349.

74. Cañaveras JC, Sánchez-Moral S, Lario J, Cuezva S, Fernández-Cortés A, Muñoz MC. El modelo de relleno, o cómo llegaron los restos a la Galería del Osario. In: de la Rasilla M, Rosas A, Cañaveras JC, Lalueza-Fox C, editors. La Cueva de El Sidrón (Borines, Piloña, Asturias). Investigación Interdisciplinar de un Grupo Neandertal. Oviedo: Consejería de Cultura y Turismo-Ediciones Trabe; 2011. pp. 147–148.

75. Wood RE, Higham TFG, de Torres T, Tisnérat-Laborde N, Valladas H, Ortiz JE, Lalueza-Fox C, et al. A new date for the Neanderthals from El Sidrón cave (Asturias, Northern Spain). Archaeometry. 2013;55: 148-158.

76. Badal-García E. La materia vegetal carbonizada. In: de la Rasilla M, Rosas A, Cañaveras JC, Lalueza-Fox C, editors. La Cueva de El Sidrón (Borines, Piloña, Asturias). Investigación Interdisciplinar de un Grupo Neandertal. Oviedo: Consejería de Cultura y Turismo-Ediciones Trabe; 2011. pp. 157-158.

77. Fortea J, de la Rasilla M, Martínez-Maza C, Sánchez-Moral S, Cañaveras JC, Cuezva S, et al. La cueva de El Sidrón (Borines, Piloña, Asturias): primeros resultados. Estud Geol. 2003;59: 159-179.

78. Rosas A, Huguet R, Estalrrich A, García-Tabernero A, García-Vargas S, Bastir M, et al. Fauna de macromamíferos en la Galería del Osarío. In: de la Rasilla M, Rosas A, Cañaveras JC, Lalueza-Fox C, editors. La Cueva de El Sidrón (Borines, Piloña, Asturias). Investigación Interdisciplinar de un Grupo Neandertal. Oviedo: Consejería de Cultura y Turismo-Ediciones Trabe; 2011. pp.147-148.

79. Sanchíz B, Martín C. La herpetofauna del yacimiento de El Sidrón. In: de la Rasilla M, Rosas A, Cañaveras JC, Lalueza-Fox C, editors. La Cueva de El Sidrón (Borines, Piloña, Asturias). Investigación Interdisciplinar de un Grupo Neandertal. Oviedo: Consejería de Cultura y Turismo-Ediciones Trabe; 2011. pp. 155-157.

80. Sesé C. Los pequeños mamíferos del yacimiento de El Sidrón. In: de la Rasilla M, Rosas A, Cañaveras JC, Lalueza-Fox C, editors. La Cueva de El Sidrón (Borines, Piloña, Asturias). Investigación Interdisciplinar de un Grupo Neandertal. Oviedo: Consejería de Cultura y Turismo-Ediciones Trabe; 2011. pp. 148-155.

81. Bœuf O. Faune et nouveaux restes humains du gisement moustérien du Petit-Puyrousseau (Charente). Mémoires de la Société Archéologique et historique de la Charente. 1969; 53-128.

82. Valladas H, Mercier N, Hovers E, Froget L, Joron JL, Kimbel WH, et al. TL dates for the Neanderthal site of the Amud Cave, Israel. J Archaeol Sci. 1999;26: 259-268.

83. Rink WJ, Schwarcz HP, Lee HK, Rees-Jones J, Rabinovich R, Hovers E. Electron Spin Resonance (ESR) and Thermal Ionization Mass Spectrometric (TIMS) 230Th/ 234U dating of teeth in Middle Paleolithic layers at Amud Cave, Israel. Geoarchaeology. 2001;16: 701-717.

84. Kolska Horwitz L, Hongo H. Putting the meat back on old bones. A reassessment of Middle Palaeolithic fauna from Amud Cave (Israel). In: Villa E, Gourichon L, Choyke AM, Buitenhuis H, editors. Archaeozoology of the Near East Proceedings of the eighth international symposium on the archaeozoology of southwestern Asia and adjacent areas. Lyon: Travaux de la Maison de l'Orient et de la Méditerranée, 49; 2006. pp. 45-64.

85. Rabinovich R, Hovers E. Faunal analysis from Amud Cave: preliminary results and interpretations. Int J Osteoarchaeol. 2004;14: 287-306.

86. Belmaker M, Hovers E. A diachronic study of the micromammal remains of Amud Cave, Israel: implications for the paleoecology of a Neanderthal site during MIS 4-3 in the Levant. Abstracts of the 73rd Society for American Archaeology Meeting. 2008:75.

87. Cheddadi R, Rossignol-Strick M. Eastern Mediterranean Quaternary paleoclimates from pollen and isotope records of marine cores in the Nile Core area. Paleoceanography. 1995;10: 291-300.

88. Behre KE, van der Plicht J. Towards absolute chronology for the last glacial period in Europe: radiocarbon dates from Oerel, northern Germany. Veg Hist Archaeobot. 1992;1: 111-117.

89. Woillard GM, Mook WG. Carbon-14 dates at Grande Pile: Correlation of land and sea chronologies. Science. 1982;215: 159-161.

90. Harvati K, Darlas A, Bailey SE, Rein TR, El Zaatari S, Fiorenza L, et al. New Neanderthal remains from Mani peninsula, Southern Greece: The Kalamakia Middle Paleolithic cave site. J Hum Evol. 2013;64: 486-499.

91. Lebreton V, Psathi E, Darlas A. Environnement végétal des néandetaliens de la grotte de Kalamakia (Arépolos, Grèce). In: Darlas A, Mihailovic D, editors. The Palaeolithic of the Balkans. Oxford: British Archaeological Reports, 1819; 2008. pp. 61-68.

92. Roger T, Darlas A. Microvertébrés, paléo environnement et paléoclimat de la grotte de Kalamakia (Péloponnèse, Grèce). In: Darlas A, Mihailovic D, editors. The Palaeolithic of the Balkans. Oxford: British Archaeological Reports, 1819; 2008. pp. 77-84.

93. Schwarcz HP, Buhay W, Grun R, Valladas H, Tchernov E, Bar-Yosef O, et al. ESR dating of the Neanderthal site, Kebara Cave, Israel. J Archaeol Sci. 1989;16: 653-659.

94. Valladas H, Joron JL, Valladas G, Arensburg B, Bar-Yosef O, Belfer-Cohen A, et. al. Thermoluminescence dates for the Neanderthal burial site at Kebara in Israel. Nature. 1987;330: 159-160.

95. Eisenmann V. Systematic and biostratigraphical interpretation of the equids from Qafzeh, Tabun, Skhul and Kebara (Acheuloyabrudian to Upper Paleolithic of Israel). Archaeozoologica. 1992 ;1: 43-62.

96. Speth JD, Tchernov E. The role of hunting and scavenging in Neandertal procurement strategies: new evidence from Kebara Cave (Israel). In: Akazawa T, Aoki K, Bar-Yosef O, editos. Neandertals and Modern Humans in Western Asia. New York: Plenum Press; 1998. pp. 223-239.

97. Speth JD, Tchernov E. Neandertal hunting and meat-processing in the Near East: Evidence from Kebara Cave (Israel). In: Stanford CB, Bunn HT, editors. Meat-Eating and Human Evolution. Oxford: Oxford University Press; 2001. pp. 52-72.

98. Speth JD, Tchernov E. Middle Paleolithic tortoise use at Kebara Cave (Israel). J Archaeol Sci 2002;29: 471-483.

99. Tchernov E. The Middle Palaeolithic mammalian sequence and its bearing on the origin of Homo sapiens In: Bar-Yosef O, Vandermeersch B, editors. Le Squelette Moustérien de Kebara 2. Paris: CNRS Cahiers de Paléoanthropologie; 1991. pp. 77-88.

100. Tchernov E. The Faunal Sequence of the Southwest Asian Middle Paleolithic in Relation to Hominid Dispersal Events. In: Akazawa T, Aoki K, Bar-Yosef O, editors. Neandertals and Modern Humans in Western Asia. New York: Kluwer Academic Publishers; 2002. pp. 77-90.

101. Debénath A, Jelinek J. Nouvelles fouilles à La Quina: Résultats preliminaires. Gallia Préhist. 1998;40: 29-74.

102. Verna C. Les restes humains moustériens de la station Amont de La Quina (Charente, France). Contexte archéologique et constitution de l’assemblage. Étude morphologique et métrique des restes crânio-faciaux. Apport à l’étude de la variation néandertalienne. Ph.D. Dissertation. Université Bordeaux 1, Talence; 2006.

103. Mercier N, Valladas H. Datations. Gallia Préhist. 1998;40: 70-71.

104. Bouchud J. Essai sur le Renne et la climatologie du Paléolithique moyen et supérieur. Périgueux: Imprimerie Magne; 1966.

105. Henri-Martin G. Découverte d'un temporal humain néandertalien dans le gisement de La Quina, Charente. C R Acad Sci. 1966;262: 1937-1939.

106. Renault-Miskovsky J. Palynologie. Gallia Préhist. 1998;40: 65-68.

107. Schwartz JH, Tattersall I. The Human Fossil Record: Terminology and Craniodental Morphology of Genus Homo (Europe). New York:Wiley-Liss; 2002.

108. Gross H. Die Umwelt des Neandertaler. In: Tackenberg K, editor. Der Neandertaler und seine Umwel. Bonn: Rheinisches Landesmuseum Bonn und Verein von Altertumsfreunden im Rheinlande; 1956. pp. 68-105.

109. Leroi-Gourhan A. Le passage Moustérien-Châtelperronien à Arcy-sur-Cure. Bull Soc Préhist Fr. 1988;85: 102-104.

110. Leroi-Gourhan A. Les Fouilles d'Arcy-sur-Cure (Yonne). Gallia Préhist. 191;4: 3-16.

111. Couture C, Tournepiche J-F. Les restes humains de la grotte de Rochelot (Charante). Anthropologie et Préhistoire. 1997;108: 99-108.

112. Tournepiche J-F, Couture C. The Hyena den of Rochelot Cave (Charente, France). Monographien des Römisch-Germanischen Zentralmuseums. 1999;42: 89-101.

113. Grün R, Stringer C. Electron spin resonance dating and the evolution of modern humans. Archaeometry. 1991;33: 153-199.

114. Mercier N, Valladas H. Reassessment of TL age estimates of burnt flints from the Paleolithic site of Tabun Cave, Israel. J Hum Evol. 2003;45: 401-409.

115. Jelinek AJ, Farrand WR, Haas G, Horowitz A, Goldberg P. New excavations at the Tabun Cave, Mount Carmel, Israel 1967-1972. Paléorient. 1973;1: 151-183.

116. Blackwell B, Schwarcz HP, Debénath A. Absolute dating of hominids and Palaeolithic artifacts of the cave of La Chaise-de-Vouthon (Charente), France. J Archaeol Sci. 1983;10: 493-513.

117. Schwarcz HP, Debénath A. Datation absolue des restes humains de La Chaise-De-Vouthon (Charente) au moyen du déséquilibre des séries d'Uranium. C R Acad Sci 1979;288: 1155-1157.

118. Fellag H. Etude palynologique de l'abri paléolithique Bourgeois-Delaunay (Chaise, Charente). Quaternaire. 1996;7: 187-196.

119. Howell FC. European and northwest African Middle Pleistocene Hominids. Curr Anthropol. 1960;1: 195-232.

120. Vallois H. La mandibule humaine pre-mousterienne de Montmaurin. C R Acad Sci Paris. 1955;240: 1577-1579.

121. Vallois H. The pre-Mousterian human mandible from Montmaurin. Am J Phys Anthropol. 1956;14: 319-323.

122. Girard M, Renault-Miskovsky J. Datation et paléoenvironnement de la mandibule de Montmaurin (Montmaurin, Haute-Garonne): analyses polliniques dans la Niche. C R Acad Sci Paris. 1983;296: 393-395.

123. Renault-Miskovsky J, Girard M. Palynologie des grottes de Montmaurin (Haute-Garonne) et du versant nord pyrénéen. Corrélations interséquentielles du Pléistocène moyen à l'Holocène. Quaternaire. 1998;9: 185-201.

124. Roebroeks W, Tuffreau A. Palaeoenvironment and settlement patterns of the Northwest European Middle Palaeolithic. In: Roebroeks W, Gamble C, editors. The Middle Palaeolithic Occupation of Europe. Leiden: University of Leiden; 1999. pp. 121-138.

125. Bahain JJ, Sarcia MN, Falguères C, Yokoyama Y. Attempt at ESR Dating of Tooth Enamel of French Middle Pleistocene Sites. Applied Radiation and Isotopes 1993;44: 267-272.

126. Huxtable J, Aitken M. Datation par thermoluminescence. In: Tuffreau A, Sommé J, editors. Le Gisement Paléolithique Moyen de Biache-Saint-Vaast (Pas de Calais). Madison: Société préhistorique française; 1988. pp. 107-108.

127. Auguste P. Etude archaéozoologique des grands mammifères du site Pleistocène moyen de Biache-Saint-Vaast (Pas-De-Calais, France): apports biostratigraphiques et palethnographiques. L'Anthropologie. 1992;96: 49-70.

128. Tuffreau A, Munaut A, Puisségur J-J, Sommé J. Stratigraphie et environnement de la séquence archéologique de Biache-Saint-Vaast (Pas-de-Calais). Bulletin de l'Association française pour l'étude du quaternaire. 1982;19: 57-61.

129. Debenham NC, Atkinson T, Grün R, Hebden N, Higham TFG, Housley RA, et al. Dating. In: Aldhouse-Green SHR, Peterson R, Walker KE, editors. Neanderthals in Wales: Pontnewydd and the Elwy Valley Caves. Oxford: Oxbow Books; 2012. pp. 283-319.

130. Aldhouse-Green S. The Pontnewydd people, their cave and their world. In: Aldhouse-Green S, Peterson R, Walker EA, editors. Neanderthals in Wales: Pontnewydd and the Elwy Valley Caves. Oxford: Oxbow Books; 2012. pp. 327-344.

131. Currant AP, Eastham A. The fauna. In: Aldhouse-Green S, Peterson R, Walker EA, editors. Neanderthals in Wales: Pontnewydd and the Elwy Valley Caves. Oxford: Oxbow Books; 2012. pp. 100-117.

132. van Asperen EN. Position of the Steinheim interglacial sequence within the marine oxygen isotope record based on mammal biostratigraphy. Quat Int. 2013;292: 33-42.

133. Adam KD. Di mittelpleistozänen Faunen von Steinheim an der Musrr (Württemberg). Quaternaria. 1954;I: 131-144.

134. Arsuaga JL, Martínez I, Arnold LJ, Aranburu A, Gracia-Téllez A, Sharp WD, et al. Neandertal roots: cranial and chronological evidence from Sima de los Huesos. Science. 2014;344: 1358-1363.

135. García-Antón M. Estudio preliminar de los yacimientos de la Sierra de Atapuerca (Burgos). In: Aguirre E, Carbonell E, Bermúdez de Castro JM, editors. El Hombre fósil de Ibeas y el Pleistoceno de la Sierra de Atapuerca. Soria: Junta de Castilla y León, Consejería de Cultura y Bienestar Social; 1987. pp. 55-59.

136. García N, Arsuaga JL. The Sima de los Huesos (Burgos, northern Spain): palaeoenvironment and habitats of Homo heidelbergensis during the Middle Pleistocene. Quat Sci Rev. 2011;30: 1413-1419.

137. Bailey SE. Neandertal dental morphology: Implications for modern human origins. Ph.D. Dissertation, Arizona State University, Tempe. 2002.

138. Freidline SE, Gunz P, Harvati K, Hublin J-J. Middle Pleistocene human facial morphology in an evolutionary and developmental context. J Hum Evol. 2012;63: 723-740.

139. Falguères C, Bahain JJ, Yokoyama Y, Bischoff JL, Arsuaga JL, Bermúdez de Castro, JM, et al. New U-series dates at the Caune de l’Arago, France. J Archaeol Sci. 2004;31: 941-952.

140. Hanquet C, Desclaux E. Analyse paléoécologique des communautés de micromammifères de la Caune de l’Arago (Tautavel, France) dans le contexte des migrations de faunes en Europe méridionale au cours du Pléistocène moyen. Quaternaire. 2011;22: 35-45.

141. Iacumin P, Cominotto D, Longinelli A. A stable isotope study of mammal skeletal remains of mid-Pleistocene age, Arago cave, eastern Pyrenees, France. Evidence of taphonomic and diagenetic effects. Palaeogeogr, Palaeoclimatol, Palaeoecol. 1996;126: 151-160.

142. Yokoyama Y, Nguyen H-V. Datation directe de l'Homme de Tautavel par la spectrométrie gamma, non destructive, du crâne humaine fossile Arago XXI. C R Acad Sci Paris. 1981;292: 741-744.

143. de Lumley H, Fournier A, Park YC, Yokoyama Y, Demouy A. Stratigraphie du remplissage Pléistocène moyen de la Caune de l'Arago a Tautavel. Etude de huit carottages effectués de 1981 a 1983. L'Anthropologie. 1984;88: 5-18.

144. de Lumley H, Grégoire S, Barsky D, Batalla G, Bailon S, Belda V, et al. Habitat et mode de vie des chasseurs paléolithiques de la Caune de l’Arago (600 000–400 000 ans). L'Anthropologie. 2004;108: 159-184.
