# Supplementary material for: Neandertal versus Modern Human Dietary Responses to Climatic Fluctuations
Source: PLoS One. 2016 Apr 27;11(4):e0153277. doi: 10.1371/journal.pone.0153277 (PMC4847867; doi:10.1371/journal.pone.0153277)
Supplement: S1 Table — (DOCX) [file pone.0153277.s002.docx]

**SI Table.**

Individual microwear data for all Paleolithic hominins included in this study. Modern human data from El Zaatari & Hublin (4), later Neandertal data from El Zaatari et al. (1-2) and Harvati et al. (3), and early Neandertal data is from this study. *Asfc*: Complexity, *epLsar*: Anisotropy, *Smc*: Scale of maximum complexity, *Tfv*: Textural fill volume, *HAsfc*: Heterogreneity.

|  | **MIS** | **Vegetation Cover** | ***Asfc*** | ***epLsar*** | ***Smc*** | ***Tfv*** | ***HAsfc*** |
| --- | --- | --- | --- | --- | --- | --- | --- |
| **Upper Paleolithic Modern Humans** | | | | | | | |
| Farincourt 1 | 2 (Magdalenian) | open | 2.695 | 0.0022 | 0.209 | 13041.8 | 0.157 |
| St Germain la Rivière 1970-7-6 | 2 (Magdalenian) | open | 3.608 | 0.0019 | 0.151 | 15737.8 | 0.157 |
| Lachaud 3 | 2 (Magdalenian) | open | 3.295 | 0.0021 | 0.150 | 10514.1 | 0.411 |
| Abri Pataud 1 | 3 (Proto-Magdalenian/ Final Gravettian) | mix | 3.564 | 0.0013 | 0.150 | 8585.5 | 0.058 |
| Isturitz 115 | 3 (Gravettain) | open | 1.420 | 0.0028 | 0.417 | 7238.5 | 0.062 |
| Dolní Věstonice 13 | 3 (Gravettain) | mix | 1.451 | 0.0009 | 0.267 | 5079.1 | 0.092 |
| Dolní Věstonice 15 | 3 (Gravettain) | mix | 1.450 | 0.0050 | 0.150 | 7997.9 | 0.110 |
| Dolní Věstonice 16 | 3 (Gravettain) | mix | 1.532 | 0.0031 | 0.150 | 14524.1 | 0.154 |
| Dolní Věstonice 31 | 3 (Gravettain) | mix | 1.150 | 0.0037 | 0.941 | 446.8 | 0.056 |
| Pavlov 1 | 3 (Gravettain) | mix | 1.984 | 0.0011 | 0.267 | 14458.6 | 0.132 |
| Předmostí 21 | 3 (Gravettain) | mix | 0.745 | 0.0033 | 0.341 | 8600.6 | 0.233 |
| Abri Labatut | 3 (Gravettain) | unavailable | 1.923 | 0.0014 | 0.342 | 979.7 | 0.019 |
| Cro-Magnon 2 | 3 (Gravettain) | open | 1.868 | 0.0039 | 0.433 | 10170.1 | 0.125 |
| Barma Grande 1 | 3 (Gravettain) | mix | 1.129 | 0.0025 | 0.341 | 2347.2 | 0.190 |
| Barma Grande 2 | 3 (Gravettain) | mix | 0.626 | 0.0042 | 262.147 | 13430.9 | 0.417 |
| Les Rois R50 #31 | 3 (Aurignacian) | open | 1.368 | 0.0020 | 0.150 | 8823.0 | 0.179 |
| Mladeč 1 | 3 (Aurignacian) | mix | 1.405 | 0.0023 | 0.267 | 1677.7 | 0.173 |
| Mladeč 2 | 3 (Aurignacian) | mix | 0.952 | 0.0026 | 0.267 | 7597.7 | 0.165 |
| Mladeč 8 | 3 (Aurignacian) | mix | 1.824 | 0.0009 | 0.208 | 6621.2 | 0.237 |
| Blanchard 1 | 3 (Aurignacian) | open | 1.625 | 0.0022 | 0.267 | 1425.4 | 0.129 |
|  |  |  |  |  |  |  |  |
| **Later Neandertals** | | | | | | | |
| Grotta Breuil 2 | 3 (<45 ka cal BP) | wooded | 2.940 | 0.0016 | 0.209 | 15454.3 | 1.032 |
| Spy | 3 (<45 ka cal BP) | open | 1.368 | 0.0032 | 0.267 | 9854.3 | 0.039 |
| Zafarraya 4 | 3 (<45 ka cal BP) | Wooded | 2.115 | 0.0051 | 0.150 | 17594.7 | 0.300 |
| Saint-Césaire 1 | 3 (<45 ka cal BP) | mixed | 2.329 | 0.0016 | 0.208 | 7687.3 | 0.435 |
| Vindija 11.45 | 3 (<45 ka cal BP) | mixed | 0.921 | 0.0026 | 0.150 | 2239.4 | 0.457 |
| Vindija 11.46 | 3 (<45 ka cal BP) | mixed | 1.165 | 0.0030 | 2.033 | 10389.0 | 0.108 |
| Vindija 12.1 | 3 (<45 ka cal BP) | mixed | 1.123 | 0.0031 | 0.267 | 17865.4 | 0.256 |
| Lakonis LKH1 | 3 (<45 ka cal BP) | wooded | 3.521 | 0.0013 | 10.507 | 13410.4 | 0.125 |
| El Sidrón SDR-005 | 3 (<45 ka cal BP) | wooded | 2.692 | 0.0022 | 7.628 | 14225.6 | 0.213 |
| Petit-Puymoyen 2 | 3 (>45 ka cal BP) | mixed | 2.070 | 0.0021 | 0.150 | 14457.9 | 0.223 |
| Petit-Puymoyen 4 | 3 (>45 ka cal BP) | mixed | 2.019 | 0.0042 | 0.267 | 17486.9 | 0.145 |
| Amud I | 3 (>45 ka cal BP) | wooded | 3.075 | 0.0013 | 0.150 | 12118.1 | 0.982 |
| Kalamakia KAL 3 | 4-3 | mixed | 1.787 | 0.0016 | 0.208 | 13744.1 | 0.076 |
| Kebara 2 | 4 | mixed | 1.547 | 0.0027 | 0.150 | 10043.2 | 0.453 |
| La Quina 5 | 4 | open | 1.176 | 0.0021 | 0.342 | 10312.9 | 0.171 |
| La Quina 20 | 4 | open | 1.168 | 0.0014 | 0.150 | 8936.2 | 0.039 |
| Subalyuk 1 | 4 | open | 0.755 | 0.0030 | 0.418 | 11573.9 | 0.239 |
| Grotte de l’Hyène IVb6 B9 | 5b | open | 0.916 | 0.0016 | 0.343 | 13174.0 | 0.072 |
| Rochelot 1098 | 5d-a | mixed | 1.610 | 0.0051 | 0.417 | 9899.9 | 0.223 |
| Tabun II | 6-5 | mixed | 1.129 | 0.0029 | 0.267 | 3351.0 | 0.22 |
| La Chaise BD8 | 6 | mixed | 1.460 | 0.0024 | 0.509 | 15933.1 | 0.076 |
|  |  |  |  |  |  |  |  |
| **Early Neandertal** | | | | | | | |
| Montmaurin – La Niche 1 | 6 | Mixed | 1.094 | 0.0038 | 2.541 | 14518.2 | 0.303 |
| Biache-Saint-Vaast 1 | 7/6 | Mixed | 1.332 | 0.0012 | 0.342 | 16674.7 | 0.135 |
| Pontnewydd PN1 | 7 | Wooded | 3.387 | 0.0023 | 0.208 | 17958.7 | 0.212 |
| Steinheim 1 | 9 or 11 | Wooded | 1.137 | 0.0042 | 1.407 | 22639.5 | 0.888 |
| Sima de los Huesos 4 | 11 | Wooded | 1.740 | 0.0016 | 0.342 | 12514.2 | 0.406 |
| Sima de los Huesos 7 | 11 | Wooded | 1.478 | 0.0016 | 31.752 | 11580.5 | 0.168 |
| Sima de los Huesos 8 | 11 | Wooded | 2.768 | 0.0038 | 0.208 | 20149.1 | 0.181 |
| Sima de los Huesos 17 | 11 | Wooded | 1.704 | 0.0024 | 6.538 | 7402.6 | 0.112 |
| Sima de los Huesos 19 | 11 | Wooded | 1.840 | 0.0031 | 7.765 | 14509.1 | 1.364 |
| Arago 13 | 10-12 | Open | 0.777 | 0.0031 | 0.267 | 15516.1 | 0.153 |
| Arago 54 | 10-12 | Open | 0.862 | 0.0021 | 52.976 | 10007.7 | 0.110 |

1. El Zaatari S, Grine FE, Ungar PS, Hublin J-J. Ecogeographic variation in Neandertal dietary habits: evidence from occlusal microwear texture analysis. J Hum Evol. 2011;61: 411-424.

2. El Zaatari S, Harvati K, Panagopoulou E. Occlusal molar microwear texture analysis: the method and its application for the dietary reconstruction of the Lakonis Neandertal. In: Voutsaki S, Valamoti S-M, editors. Subsistence, Economy and Society in the Greek World. Louvain: Peeters Publishers; 2013. pp. 55-63.

3. Harvati K, Darlas A, Bailey SE, Rein TR, El Zaatari S, Fiorenza L, et al. New Neanderthal remains from Mani peninsula, Southern Greece: The Kalamakia Middle Paleolithic cave site. J Hum Evol. 2013;64: 486-499.

4. El Zaatari S, Hublin J-J. Diet of Upper Paleolithic modern humans: evidence from microwear texture analysis. Am J Phys Anthropol. 2014;153: 570-581.
